# Gene and transposable element expression in response to stress in temperate and tropical populations of *Drosophila*

**DOI:** 10.1186/s13100-025-00372-x

**Published:** 2025-09-30

**Authors:** Miriam Merenciano, Daniel S. Oliveira, Judit Salces-Ortiz, Rita Rebollo, Bianca Manfré, Bianca Menezes, Gabriel Krasovec, Camille Simonet, Sonia Janillon, Nelly Burlet, Claudia M. A. Carareto, Cristina Vieira, Marie Fablet

**Affiliations:** 1 Laboratoire de Biométrie et Biologie Évolutive, Université Claude Bernard Lyon 1, CNRS, UMR5558, Villeurbanne, Rhône-Alpes 69100 France; 2 Institute of Biosciences, Humanities and Exact Sciences, São Paulo State University (Unesp), São José do Rio Preto, São Paulo, 15054-000 Brazil; 3 Univ Lyon, INRAE, INSA-Lyon, BF2I, UMR 203, Villeurbanne, 69621 France; 4 Federal Institute of Rio de Janeiro (IFRJ), Duque de Caxias, RJ Brazil; 5 Institut Universitaire de France (IUF), Paris, Île-de-France F-75231 France; 6 Present Address: Institute of Evolutionary Biology, CSIC, UPF, Barcelona, 08003 Spain; 7 Present Address: Université Paris Cité, CNRS, Institut Jacques Monod, Paris, F- 75013 France

**Keywords:** *Drosophila*, Stress, Transposable elements, Transcriptome, Epigenetics

## Abstract

**Background:**

The study of stress response in natural populations is crucial for understanding species local adaptation and evolution. In *Drosophila*, significant genetic diversity across populations from different geographical origins has been observed, emphasizing the influence of local environments.

**Results:**

In this study, we explored the impact of starvation and cold stress on the phenotypic and transcriptomic response of two natural populations of *D. melanogaster* and *D. simulans* from temperate and tropical regions. Additionally, we investigated the behavior and influence of transposable elements (TEs) in these types of stress, combining RNA-seq and ChIP-seq experiments, with high-quality long-read genome assemblies of all the strains. Our findings in *D. melanogaster* revealed that the transcriptomic response to stress is similar across geographical origins, whereas in *D. simulans* there is more variability. Notably, neither starvation nor cold induced a general activation of TEs in *D. melanogaster* or *D. simulans*, at least in the tissue and strains analysed in this study. Finally, we found three polymorphic TEs producing TE-chimeric transcripts associated with changes in nearby gene expression levels after stress.

**Conclusions:**

Overall, this study highlights the complexity of stress-TE interactions and their potential impact on adaptation. Understanding these dynamics contributes to the broader knowledge of how genetic and environmental factors interact to modulate gene expression, shaping an organism’s ability to adapt to varying conditions.

**Supplementary Information:**

The online version contains supplementary material available at 10.1186/s13100-025-00372-x.

## Background

Studying the response to stress in different natural populations is of paramount importance in understanding the adaptation and evolution of species. Different stress types, biotic or abiotic, pose significant challenges to the survival and well-being of organisms. By examining how various populations react to these stressors, it is possible to gain insights into the mechanisms underlying adaptation and evolution, and to understand the ecological dynamics and potential impacts of global environmental changes.

In *Drosophila* species, natural populations with different geographical origins often exhibit striking genetic diversity, further emphasizing the role of local environments and selective pressures in shaping their genetic landscape [1–9]. For instance, recent studies in *Drosophila melanogaster* have shown higher levels of genetic diversity for the ancestral African populations, compared to North American and European populations [4, 7, 8]. In addition, the phenotypic response to different types of stress has been extensively studied in different natural populations, showing inter- and intra-population differences [10–15]. However, although the transcriptomic response to different types of stress such as oxidative stress, infection, heat, and desiccation, has been reported, there is little information on the transcriptomic response to stress in natural populations of different climate origins [16–23].

Starvation and cold are two physiological stressful conditions that *D. melanogaster* and its sister species *D. simulans* experience in temperate areas where seasonal fluctuations in food availability and temperature are pronounced [24, 25]. Previous studies comparing natural populations from temperate and tropical regions found that resistance to starvation tends to decrease with latitude, with tropical populations showing greater resistance [26–30]. However, no clinal patterns were observed in *D. melanogaster* collected in South America, suggesting that selection on starvation resistance may be inconsistent across continents [29, 31]. Regarding cold stress, *D. melanogaster* flies from tropical regions were found to be less resistant to cold compared to their temperate counterparts [28, 32–34]. This correlation, however, was not observed in *D. simulans*, where temperate populations did not have higher levels of thermal plasticity when facing cold stress compared to tropical ones [35]. Although the phenotypic response to both starvation and cold stress has already been studied between populations from different climate regions, the comparison of the transcriptomic response between populations and also between species is still lacking.

Transposable elements (TEs) are repetitive sequences capable of moving within the genome and have been described to play an important role in stress response [36–41]. Stress can lead to changes in TE-silencing mechanisms, such as DNA methylation and histone modifications, as well as alterations in the expression of small non-coding RNAs [40]. When TE-silencing mechanisms are disrupted, TEs can be activated and potentially induce genomic instability with neutral, harmful or even beneficial effects on the genome [40, 42]. In fact, stress-activated TEs can serve as a source of raw genetic material for the generation of new genes or the spread of regulatory elements that can rewire stress response networks [43, 44]. However, the link between stress and TEs is complex [40]. TEs are not always activated by stress, and examples of both activation and repression have been reported in *Drosophila* under several stress conditions such as oxidative stress, viral infection, and ionizing radiation [45–49]. Yet, information is lacking regarding the analysis of TE activation and/or repression and its role in the organismal transcriptomic response to starvation and cold stress in both *D. melanogaster* and *D. simulans*. These stressors are ecologically relevant and their study would allow to examine how different environmental challenges can influence TE regulation. Moreover, the study of stress effects on both TE expression and epigenetic landscape would provide a better understanding on how genetic and environmental factors interact to modulate gene expression, ultimately influencing an organism’s adaptability.

TEs can also contribute to transcriptome diversity by generating gene-TE chimeric transcripts, which are mRNA molecules with both gene and TE-derived sequences [50–54]. In *D. melanogaster*, the generation of chimeric transcripts has been studied in different natural populations and also in different body parts, but it has never been studied genome-wide in stress conditions [55–58]. In addition, a parallel analysis to investigate how TEs can differentially contribute to the transcriptomic diversity between closely related species has never been explored.

In this work, we studied the effect of two different stressful environmental conditions (starvation and cold stress) on phenotypic and transcriptomic variation (both genes and TEs) of two natural populations of *D. melanogaster* and *D. simulans* from temperate and tropical areas. We found that, in *D. melanogaster,* the transcriptomic response to stress is associated with the phenotypic performance rather than the geographical origin of the strains. In other words, the transcriptomic response to stress seems to be conserved across divergent populations adapted to contrasted environments. In *D. simulans* we observed that the transcriptomic response to stress differs between populations only in starvation conditions. Moreover, we found that cold stress is associated with chromatin changes in TE flanking regions. Finally, despite the fact that neither the global TE expression nor the chimeric transcript production was affected by stress, we identified three TEs producing strain-specific chimeric transcripts associated with changes in nearby gene expression levels.

## Methods

### Drosophila strains and rearing

We used nine inbred strains of *D. melanogaster* and nine inbred strains of *D. simulans* from Menezes et al., (2018) [59] (Table S1). Briefly, samples of natural populations of *D. melanogaster* and *D. simulans* were collected from two different geographical locations: France (Gotheron, 44º56’0”N 04º53’30”E - “goth” strains) and Brazil (São José do Rio Preto 20°41’04.3"S 49°21’26.1"W - “sj” strains) in June 2014 using fruit baits [59–61] (Table S1). Isofemale strains per species and geographical origin were established directly from gravid females from the field. Then, brother-sister crosses were subsequently performed during 30 generations, resulting in strains with very low amounts of intra-strain genetic variability [62] (Table S1). Flies were kept at 25 °C in vials with nutritive medium (0.2 g agar, 1.46 g maize flour, 1.53 g yeast, 0.08 g Nipagine, 0.63 mL ethanol, 12 mL water) with a 12:12 h light/dark cycle.

### Starvation assay

We performed adult fly survival in starvation conditions in nine inbred strains of *D. melanogaster* and nine inbred strains of *D. simulans* (Table S1). Ten two-to-five day-old adult flies were placed without anesthesia (using an insect aspirator) in vials with a non-nutritive medium composed of agar and water. 15 replicates were made per sex and strain, resulting in a total of 150 females and 150 males tested per strain. Dead flies were recorded every 6 h (Table S2). The experiment was performed at 25 °C and at a constant humidity of 60%. Mean lifespans in starvation conditions were calculated for every strain and sex separately and log-transformed using the *mean* and *log* functions in R software. Finally, a linear hierarchical mixed model was fitted to the log-transformed means for each species separately (model: lifespan ~ population/factor(strain) + (1|replicate) + sex + strain: sex) and p-values were estimated in each species using an ANOVA test with the R function *aov.* For transcriptomic and epigenomic analyses, 30 three-to-five day-old females of each strain were placed in vials with the same non-nutritive medium for 24 h and were allowed to recover in vials with nutritive medium 24 h before ovary dissection. Two replicates per strain were produced. Simultaneously, the same number of flies was also kept in vials with a nutritive medium for 48 h as controls before ovary dissection (Table S1).

### Chill coma recovery time assay

We performed chill coma recovery time (CCRT) assays in the same nine inbred strains of *D. melanogaster* and nine inbred strains of *D. simulans* mentioned before (Table S1). 48 two-to-five day-old female and male flies per strain were transferred without anesthesia (using an insect aspirator) into 48-well plates, one fly per well, and placed in chambers containing melting ice (0 °C) for 16 h. Then, individuals were removed from the cold and returned to room temperature (24 °C). Three replicates of 48 flies were made per sex and strain, resulting in a total of 144 females and 144 males tested by strain. CCRT was measured individually by recording the time until each individual could stand on its legs [63] (Table S3). CCRT measurements were log-transformed using the *log* function in R. Finally, a linear hierarchical mixed model was fitted to the log-transformed values (model: recovery time ~ population/factor(strain) + (1|replicate) + sex + strain: sex) for each species separately and p-values were estimated in each species using an ANOVA test with the R function *aov*. For transcriptomic and epigenomic analyses, 30 two-to-five day-old females of each strain were also placed for 16 h in chambers containing melting ice (0 °C) and were allowed to recover for 2 h before ovary dissection. Two replicates per strain were produced. Simultaneously, the same number of flies was also kept at room temperature (24 °C) for 18 h as controls before ovary dissection (Table S1).

### Longevity assay

For each strain, 50 recently emerged males (less than 8 h old) and 50 virgin females were collected and divided into groups of 10, placed in tubes containing nutritive medium (Table S1). Each tube was treated as an independent biological replicate, resulting in five replicates per sex and strain. Flies were separated by sex without anesthesia using an insect aspirator. Mortality was recorded daily, excluding weekends, until all individuals in a tube had died. Surviving flies were transferred to fresh food vials weekly. Longevity assays were conducted at 25 °C and a constant relative humidity of 60%.

### RNA extraction for RNA-seq analysis

Total RNA was extracted from a pool of 30 ovaries of three-to-five day-old females of every strain and experimental condition (see previous sections for information on how flies were exposed to different stress conditions). Two replicates per strain and experimental condition (control, starvation and cold) were performed, obtaining a total of 24 samples per species. We extracted RNA from ovaries because we were interested in the effects that may have an evolutionary impact and therefore potentially be transmitted to the next generation. RNA extraction was carried out using the RNeasy Plus (Qiagen) kit following manufacturer’s instructions. After DNAse treatment (Ambion), quality control was performed using an Agilent Bioanalyzer. Libraries were constructed from mRNA using the Illumina TruSeq RNA Sample Prep Kit following manufacturer’s recommendations. Libraries were sequenced on Illumina HiSeq 3000 with paired-end 150 nt reads. All RNA-seq libraries were trimmed with *Trimmomatic v0.39* [64]. Sequence files have been deposited in NCBI SRA under the accession number PRJNA1029344.

### RNA-seq mapping and quantification

RNA-seq reads from *D. melanogaster* and *D. simulans* were mapped with *STAR v.2.7.6* using default parameters [65, 66] against the Flybase *D. melanogaster* (r6.16) and *D. simulans* reference genomes (r2.02), respectively. Then, we used the alignments to quantify gene expression with *htseq v.1.99.2*, parameter *-t exon*, which uses the sum of the reads aligned in all exons to assign counts for each gene [67]. The quantification of TE expression at the family level was performed with *TEtools* using the TEcount module [68]. We used a merged dataset with TE sequences from *D. melanogaster* and *D. simulans* containing 33,086 insertions from 237 TE families available at https://pbil.univ-lyon1.fr/datasets/Roy2019 [47]. The total counts generated from the TEcount module account for uniquely mapped reads, based on the best alignment score. Therefore, multi-mapped reads are counted a single time. In cases of equal alignment scores, such as perfect marches to multiple TE copies, the read is assigned randomly.

### Differential expression analysis of genes and TEs

Differential expression analyses of genes and TEs were performed using the R package *DESeq2 v.1.40.2* [69]. The raw read counts obtained for genes and TEs were merged in a single matrix for each species, and then submitted to normalization with default parameters. This step ensures robust and stable normalization, avoiding biases that could arise from a small TE-only subset such as limiting size factor estimations leading to biases in the dispersion of transcriptome variability between samples. Differentially Expressed Genes (DEGs) and Differentially Expressed (DE) TE families were identified between control and treatments (starvation and cold) by strain, with the following model: ~strain + treatment + strain: treatment. The log2 fold-change was shrunken with *apeglm v.1.22.1* algorithm from *lfcshrink* function to remove noise and preserve large differences, improving fold-change estimations [70]. Multiple testing correction was applied using the Benjamini-Hochberg procedure, as implemented in *DESeq2*, and genes and TE families were considered significantly differentially expressed if they had an adjusted p-value (False Discovery Rate, FDR) < 0.05 and |log2 fold-change| >1 (Table S4). To test whether variation in transcriptomic response to starvation was associated with variation in stress resistance across strains, we performed a Mantel test comparing pairwise distances in gene expression (log₂ fold change between control and stress) with pairwise differences in survival time. We constructed a matrix of Euclidean distances between strains based on these fold changes, considering only genes with an absolute log₂FC > 1 in at least one strain. A corresponding distance matrix was built from the differences in mean survival time or mean CCRT under starvation and cold stress, respectively. The functional analysis of up- and down-regulated genes was performed separately with DAVIDGO, selecting Gene Ontology (GO) terms with correction by FDR < 0.05 (Table S5) [71].

### Analysis of TE insertion enrichment near DEGs

Permutation tests were performed by intersecting TE positions with gene regions (+/- 1 kb) using the R package regioneR, parameter *randomization = resampleRegions*, with 1,000 random samples [72]. Hence, for every up- and down-regulated gene set of size *n*, the software determines the count of those containing TEs in the +/- 1 kb flanking regions. Subsequently, it conducts 1,000 random samples of *n* genes each to assess if the observed number of genes with TEs deviated from the expected count in a genome-wide context.

### Chromatin immunoprecipitation

Chromatin immunoprecipitation was performed using 50 ovaries dissected from three-to-five day-old females of every strain that were previously exposed to cold (0 °C) for 16 h and following a 2 h of recovery. Simultaneously, the same number of ovaries were dissected from flies kept at room temperature (24 °C) for 18 h as controls. Two biological replicates of each strain and experimental condition were generated. Ovaries were then re-suspended in 500 µL of A1 buffer (60 mM KCl, 15 mM NaCl, 15 mM Hepes, 0,5% Triton and 10 mM sodium butyrate) and cross-linked in formaldehyde (Sigma) at a final concentration of 1.8% for 10 min at room temperature (25 °C). After, ovaries were homogenized in a 2 mL glass tissue grinder (Dounce B) 70 times. Formaldehyde was subsequently quenched using glycine (0.125 M). Cross-linked cells were washed and pelleted twice with buffer A1, once with cell lysis buffer (140 mM NaCl, 15 mM Hepes, 1 mM EDTA, 0.5 mM EGTA, 1% Triton X100, 0.1% sodium deoxycholate, 10 mM sodium butyrate), followed by lysis in buffer containing 140 mM NaCl, 15 mM Hepes, 1 mM EDTA, 0.5 mM EGTA, 1% Triton X100, 0.5% SDS, 0.5% N-Laurosylsarcosine, 0.1% sodium deoxycholate, 10 mM sodium butyrate for 120 min at 4 °C. After that, lysates were sonicated in a Bioruptor sonicator to reach a fragment size window of 200–600 bp. We then separated 20 µL of the chromatin as input and stored it at 4 °C. The remaining 180 µL were divided into two aliquots (one for each antibody used). Immunoprecipitation was done with the Magna ChIP A/G Chromatin Immunoprecipitation Kit (Merck) following manufacturer’s instructions. Chromatin was incubated overnight at 4 °C with the following antibodies: for H3K4me3 using α-H3K4me3 (millipore #07-473, 3 µg/IP) and for H3K9me3 using α-H3K9me3 (active motif #39161, 3 µg/IP) antibodies. After incubation, beads were washed with: Low salt buffer, High salt buffer, LiCl complex buffer, and TE buffer. Chromatin from IPs was eluted in 0.5 mL of elution buffer. Proteinase K was added to IP and chromatin inputs and incubated at 65 °C in a shaker at 300 rpm overnight. Then, chromatin was purified with phenol: chloroform using MaXtract High Density tubes (Qiagen) to maximize DNA recovery. Samples were eluted in 20 µL of DEPC water and stored at -20 °C until further processed. Libraries were prepared using Diagenode’s MicroPlex Library kits. Input and immunoprecipitated H3K4me3 and H3K9me3 libraries were sequenced on an Illumina HiSeq 4000 with paired-end 100 bp reads. Sequence files have been deposited in NCBI SRA under the accession number PRJNA1029344.

### ChIP-seq analysis

ChIP-seq reads underwent quality control, mapping, and differential analysis via the *nf-core/chipseq* pipeline *v2.0.0* [73]. *Trim Galore v0.6.7* was used for adapter trimming. ChIP-seq reads were then mapped against the *FlyBase D. melanogaster* (r6.16) (https://ftp.flybase.net/genomes/Drosophila_melanogaster/dmel_r6.16_FB2017_03/fasta/dmel-all-chromosome-r6.16.fasta.gz*)* and *D. simulans* (r2.02) *(*https://ftp.flybase.net/genomes/Drosophila_simulans/dsim_r2.02_FB2017_04/fasta/dsim-all-chromosome-r2.02.fasta.gz*)* reference genomes using *bowtie2 v2.4.4* with default parameters [74]. An exception was made for the *D. simulans* H3K4me3 dataset, where a corrupted R1 file led to the use of R2 reads as single-reads. Effective genome size was calculated with default parameters using *unique-kmers.py v2.1.1*. For *D. melanogaster*, reads mapping to blacklisted regions (obtained through the *nf-core/chipseq* pipeline *v2.0.0*) were removed with *bedtools v2.30.0* [75]. Duplicated reads were removed with *Picard v2.27.4-SNAPSHOT* [76] with MarkDuplicates REMOVE_DUPLICATES = TRUE, and, uniquely mapped reads were kept using *samtools v1.15.1*.

### Profiling of histone marks in TE flanking regions

Following alignment of Chip-seq reads with *bowtie2* against strain-specific genomes, we filtered duplicated reads with *sambamba*, and used the function *multiBamSummary* from *deeptools* to compute row counts of the +/- 2 kb regions of TE insertions [76, 77]. Total reads aligned in each sample were computed with *samtools view -c -f 0 × 2*, and FPKM values for each 2 kb window were generated with the formula (raw counts * 1e9) / (total counts * 2,000). FPKM values from the two biological replicates were averaged, and flanking regions with FPKM < 10 in both control and cold conditions were removed. Finally, the non-parametric Wilcoxon signed-rank test (alpha = 5%) was used to assess global chromatin differences between control and cold.

### Profiling of histone marks in full-length TE flanking regions

To assess histone enrichment at the flanking regions of full-length (FL) TE insertions in *D. melanogaster* and *D. simulans*, we first identified such insertions in each genome (Table S5). To do so, we used *RepeatMasker v.4.1.2* to mask the genomes with consensus TE sequences available at https://github.com/bergmanlab/drosophila-transposons/, and parameters *-cutoff 250*,* -a*,* -s*,* -norna*. We calculated the proportional length of each copy with the respective length of its consensus. All insertions with > = 80% of the consensus length were considered as FL copies. Since we were interested in insertional polymorphism in the wild-type strains, ChIP-seq reads were mapped against their respective wild-type genomes with *bowtie2* using default parameters. The alignments were filtered with *sambamba* to remove multi-mapped reads with parameters *view -h -t 16 -f bam -F “[XS] = = null and not unmapped and not duplicate”*. Finally, we normalized the alignments with *deeptools v.3.5.3*, function *bamCoverage*, parameters *–normalizeUsing RPGC –extendReads*, and *--effectiveGenomeSize* with the respective genome size of each species. Once we obtained FL TEs in each wild-type strain genome, as well as normalized ChIP-seq alignment depths, we used the functions *computeMatrix* (parameters *–regionBodyLength 0*, *–beforeRegionStartLength 2000*, *–afterRegionStartLength 2000*), and *plotProfile* from *deeptools* to analyse H3K4me3 and H3K9me3 enrichments.

### Small RNA extraction, sequencing and analyses

Small RNA extraction, sequencing, and analyses dedicated to TEs in control conditions had already been performed and described in Mohamed et al., (2020) [60]. The corresponding sequence files had been deposited in NCBI SRA under the accession number PRJNA644327. Small RNA extraction and sequencing in cold conditions were performed at the same time as controls, and data has been deposited in NCBI SRA under the accession number PRJNA1029344. Small RNA read counts corresponding to TEs were obtained on 23–30 nt reads using the same reference sequences as for RNA-seq analyses with the TEcount module of *TEtools* [68]. 19–39 nt reads were aligned to dmel-all-miRNA-r6.16.fasta available from *FlyBase*, and the sums of the counts corresponding to miRNAs were used to normalize data, same as in Roy et al. (2020) [47].

### Detection of chimeric transcripts

Gene-TE chimeric transcripts were identified with *ChimeraTE* Mode 1 for each condition separately [56]. Briefly, each strain-specific genome and gene/TE annotation was used to perform the alignment of RNA-seq reads. Genome assemblies were produced in Mohamed et al., (2020) [60] and were deposited in the European Nucleotide Archive (ENA) (https://www.ebi.ac.uk/ena/browser/view/PRJEB50024). Gene and TE annotations were deposited at Zenodo repository (10.5281/zenodo.10214080). Briefly, *ChimeraTE* detects paired-end reads spanning from TE insertions to exons. Since the reads were produced from mRNA molecules, these pairs with one mate aligning to the exon, and another one to the TE provide clear evidence of co-transcription between both entities, regardless of the inner sequence (sequence between read mates). Based upon the TE position in respect to genes, TE-chimeric transcripts are classified into three categories: TE-initiated when the TE is located upstream of the gene; TE-exonized when the TE is located within the gene body, either in exons or introns; and TE-terminated when the TE is located downstream of the gene. We have used default parameters, including only TEs located up to 3 kb near genes, and maintaining only TE-chimeric transcripts that have been found in both RNA-seq replicates (Table S7).

Polymorphic TE insertions generating TE-chimeric transcripts were identified between genomes of strains from the same species. To do so, we first created three gene files (*bed* format) containing the position for genes generating TE-chimeric transcripts: 3 kb upstream, gene body, and 3 kb downstream. Then, for each strain-specific genome, we intersected these regions with its respective TE annotation (*bed* file) using *bedtools v.2.30.0*, function *intersect* [76]. Therefore, for each strain and genomic region (3 kb upstream, gene body, or 3 kb downstream), we generated a list containing the gene ID and the respective TE family found. Finally, pairwise comparisons identified polymorphic TE insertions, considering a TE as polymorphic when a given gene has a TE in one of its three regions, but absent in at least another strain. Manual curation of polymorphic insertions was performed with Interactive Genomics Viewer (IGV) [78].

## Results

### Natural phenotypic variability to starvation and cold stress in different populations of two *Drosophila* species

To assess phenotypic variability in response to stress between flies from temperate and tropical climates, we measured survival after starvation and chill coma recovery time (CCRT) in nine *D. melanogaster* and nine *D. simulans* strains from two different natural populations: one from a temperate climate (France) and one from a tropical one (Brazil).

**Fig. 1:**
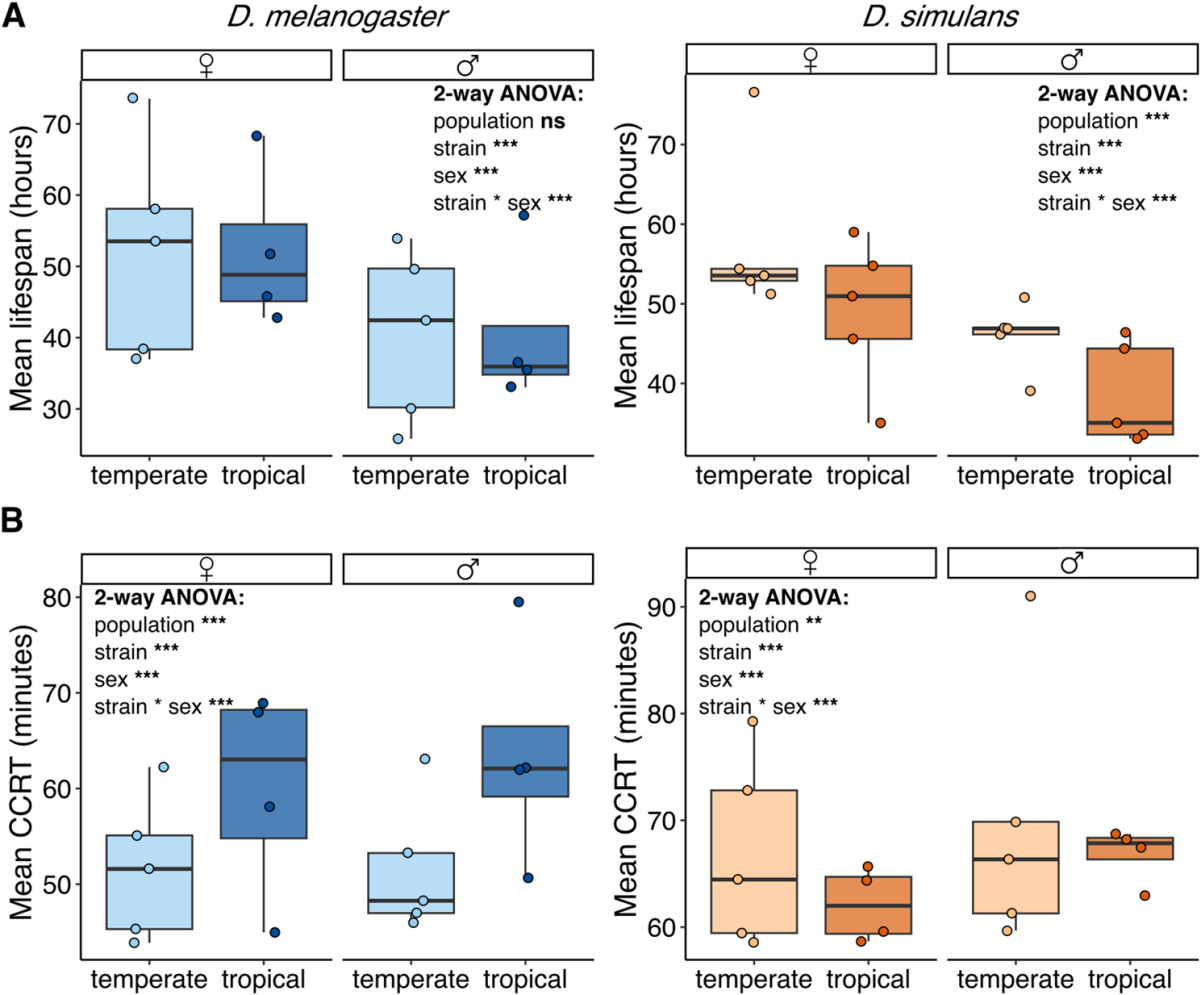
Natural variability to starvation and CCRT in *D. melanogaster* and *D. simulans*. **A**) Boxplot showing the mean lifespan in hours for each of the tested strains under starvation conditions for both *D. melanogaster* and *D. simulans* females and males. **B**) Boxplot showing the mean CCRT in minutes for each of the tested strains under cold stress conditions for both *D. melanogaster* and *D. simulans* females and males

In *D. melanogaster*, although there were no differences in starvation resistance between populations (2-way ANOVA, *p* = 0.234), ANOVA analysis showed that the effects of the strain, sex, and the interaction between these two factors were statistically significant (2-way ANOVA, p-values < 0.001) (Fig. 1A and Fig. S1A). Indeed, mean lifespans in starvation ranged from 25.8 to 57.2 h in males, and from 36.9 to 73.5 h in females, showing that females were more resistant to starvation [79, 80] (Fig. 1A and Fig. S1A). These values are markedly lower than the typical lifespans observed under nonstress conditions (Fig. S2). In *D. simulans*, similar to *D. melanogaster*, the effects of the strain, sex, and the interaction between these two factors were statistically significant (2-way ANOVA, p-values < 0.001) (Fig. 1A and Fig. S1A). However, in this species, the origin of the population did have a statistically significant effect (2-way ANOVA, *p* < 0.001), suggesting that strains from France (temperate climate) tend to be more resistant to starvation than the ones from Brazil (tropical climate) (Fig. 1A and Fig. S1A). Mean lifespans in starvation ranged from 33.1 to 50.8 h in males, and from 35.0 to 76.6 h in females, showing again that females were more resistant to starvation compared to males, and that starvation affects lifespan (Fig. 1A and Fig. S1A and S2).

We then used the same strains to assess variability to CCRT (Fig. 1B). Since we are measuring recovery time, lower values indicate greater tolerance to chill coma, and higher values indicate reduced tolerance. The effects of the strain, sex, and the interaction between these two factors were statistically significant in both species (2-way ANOVA, all p-values < 0.001), as the population origin (2-way ANOVA, *p* < 0.001 in *D. melanogaster* and *p* = 0.002 in *D. simulans*) (Fig. 1B and Fig. S1B). Indeed, temperate strains seemed to be more resistant to cold. Mean CCRT values in *D. melanogaster* ranged from 45.0 to 79.2 min in males, and from 42.9 to 67.4 min in females; whereas in *D. simulans*, mean CCRT values ranged from 57.7 to 91.0 min in males, and from 57.0 to 78.3 min in females (Fig. 1B and Fig. S1B) [79].

Furthermore, we tested whether there are differences in starvation and cold response between *D. melanogaster* and *D. simulans*. We found that the difference between species in the response to starvation was statistically significant only in females (2-way ANOVA, *p* = 0.058 in males and *p* = 0.008 in females) (Fig. S3A). For CCRT, differences between species were found both in males and females (2-way ANOVA, p-values < 0.001 in males and females), where *D. melanogaster* recovers earlier from the cold exposure compared to *D. simulans* (Fig. S3B).

Overall, we found phenotypic variation in starvation and CCRT between different natural populations of *D. melanogaster* and *D. simulans*, and that population origin had an effect on CCRT, and on starvation only in *D. simulans* (Fig. 1). For the following analysis, we focused on two temperate and two tropical strains of each species that showed extreme phenotypic responses in starvation and/or CCRT experiments in females: dmgoth101, dmgoth63, dmsj23, and dmsj7 for *D. melanogaster*, and dsgoth31, dsgoth613, dssj27, dssj9 for *D. simulans* (Fig. S1).

### Transcriptomic response to starvation tends to be similar across divergent populations in *D. melanogaster*, whereas it shows variation in *D. simulans*

To investigate whether there were population differences in the transcriptional response to starvation in the two *Drosophila* species, we used RNA-seq from ovaries of females exposed to 24 h of starvation (see Methods).

**Fig. 2:**
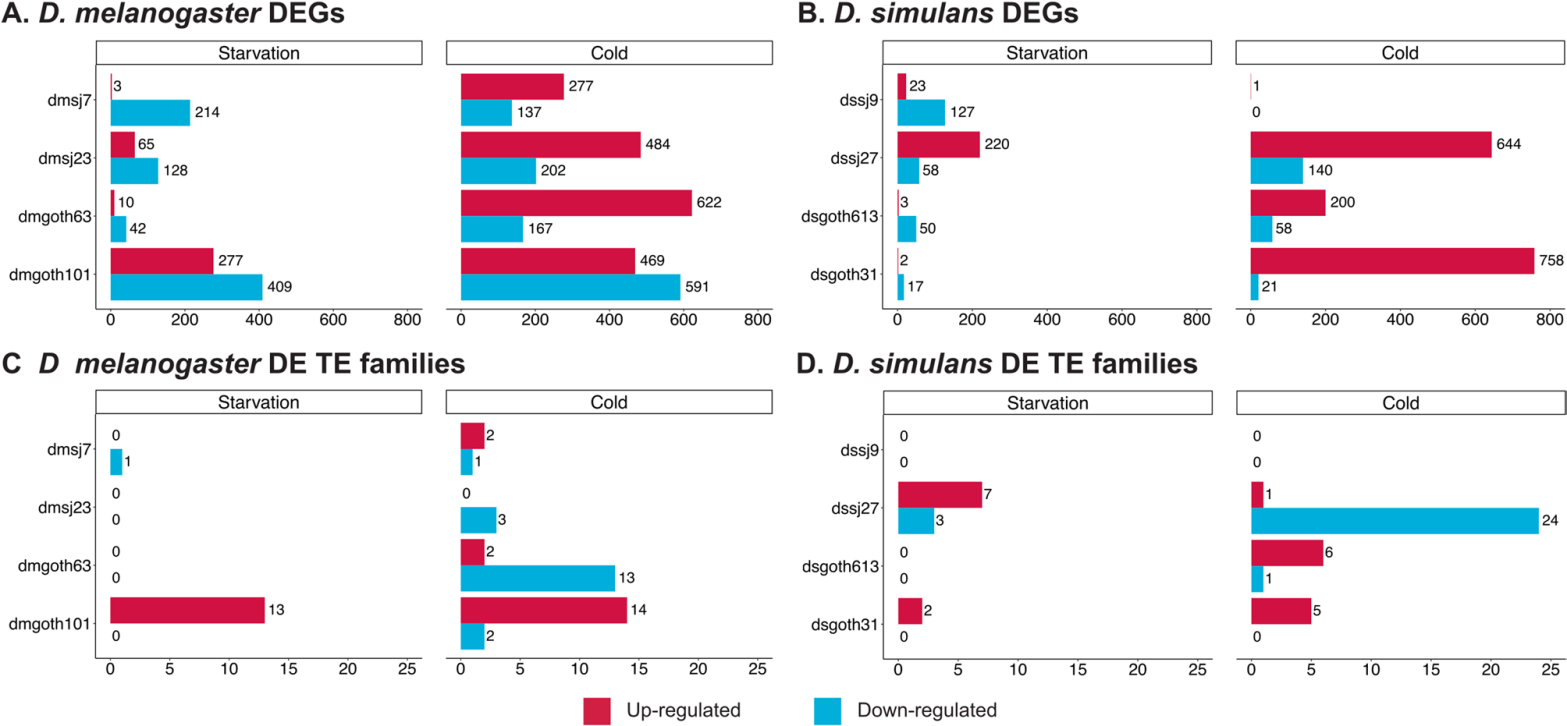
DEGs and differentially expressed TE families in response to starvation and cold stress. **A**) Number of up-regulated (red) and down-regulated (blue) genes in response to both starvation and cold stress in *D. melanogaster ***B**) and in *D. simulans* strains. **C**) Number of up-regulated (red) and down-regulated (blue) TE families in response to both starvation and cold stress in *D. melanogaster* **D**) and in *D. simulans* strains. Strains were named based on their location of origin: temperate strains are named “goth” (from Gotheron, France), while tropical strains are named with “sj” (from São José do Rio Preto, Brazil). DEG: differentially expressed genes. DE: differentially expressed

In *D. melanogaster*, a principal component analysis (PCA) of normalized gene counts under control conditions showed that PC1 (accounting for 50% of the variance) grouped strains based on their phenotypic response (more sensitive *vs.* more resistant) and PC2 (27% of the variance) grouped them by population (Fig. S4A). When considering samples exposed to starvation, PC1 (42% of the variance) separated control samples from those exposed to starvation, but only for the strains dmgoth101 and dmsj7, which exhibited increased starvation resistance. PC2 (14% of the variance) continued to group samples by population origin (Fig. S5A and S6A). To further test whether variation in transcriptomic response to starvation was associated with variation in starvation resistance across strains, we performed a Mantel test comparing pairwise distances in gene expression (log₂ fold change between control and starvation) with pairwise differences in mean survival time. Consistent with the PCA analysis, the Mantel test, based on Spearman’s rank, yielded a Mantel statistic of *r* = 0.314 with a p-value of 0.208. While this correlation was not statistically significant, likely due to the limited number of strains, it suggests a moderate association between transcriptional and phenotypic responses. The number of differentially expressed genes (DEGs) after starvation in *D. melanogaster* strains ranged from 52 to 686, comprising 0.29–3.86% of the total genes respectively, with no evident differences based on the geographic origin (Fig. 2A). Significant overlap between the DEGs from this work and previous transcriptomic analysis upon starvation was found, suggesting a consistent response triggered by starvation (Fig. S7A) [81]. All strains exhibited a higher number of down-regulated compared to up-regulated genes (Fig. 2A). Furthermore, we found that the transcriptomic response to starvation was strain-specific, as 98% (342) of the up-regulated genes and 68% (397) of the down-regulated ones were found in a strain-specific manner (Fig. S8A). Notably, the two strains that showed increased resistance to starvation (dmgoth101 and dmsj7) had a higher amount of shared DEGs, totaling 133 genes, and further suggesting an association between transcriptional and phenotypic responses (Fig. S8A). Regarding the function of the DEGs, gene ontology analysis (GO) revealed that strains that were more sensitive to starvation had an enrichment in genes related to proteolytic processes, a biological process previously linked to starvation (Table S5) [82].

In *D. simulans*, PCA analysis showed no clear sample clustering under control conditions (Fig. S4C). However, upon starvation, PC1 (32% of the variance) separated control samples from those exposed to starvation only for strains dsgoth613 and dssj9. This separation could not be associated with their phenotypic response or population origin (Fig. S5C and S6C). Moreover, although we obtained a Mantel statistic of *r* = 0.314 with a p-value of 0.417, suggesting a moderate but not statistically significant association between transcriptional and phenotypic responses, this association appears complex. As mentioned before, PCA of the transcriptomic profiles did not separate strains according to their survival phenotypes, indicating that the relationship between gene expression changes and starvation resistance is likely noisy and multifactorial. In the same species, the number of DEGs after starvation ranged from 19 to 278, representing 0.12% and 1.80% of the total genes respectively, with the two strains from Brazil having more DEGs, thus suggesting an increased transcriptomic response in the tropical population (Fig. 2B). Similar to *D. melanogaster*, we found an increased number of down-regulated genes compared to up-regulated ones, except for the tropical strain dssj27 (Fig. 2B). Again, the transcriptomic response to starvation seems to be strain-specific, as 99% (242) of the up-regulated genes and 73% (140) of the down-regulated genes are found in a strain-specific manner (Fig. S8B). dsgoth613 and dssj9 strains shared the highest number of DEGs, 30 down-regulated genes (Fig. S8B). However, these two strains displayed contrasted phenotypes upon starvation (Fig. S1A). Considering all the strains together, no common GO biological processes were found between the strains, where DEGs were involved in processes such as signaling, transport, as well as eggshell formation (Table S5).

In summary, we found that *D. melanogaster* transcriptomic response to starvation was first determined by the phenotypic response and then by the geographical origin of the population. However, in *D. simulans*, strains from Brazil (tropical) had more pronounced gene expression changes compared to strains from France (temperate). These results might indicate that, although the transcriptomic response to starvation appeared to be strain-specific, it was consistent across different geographical origins in *D. melanogaster*. In contrast, in *D. simulans*, we observed less consistency, likely suggesting that the transcriptomic response to starvation differs between populations in this species.

### Transcriptomic response to cold is not associated with the geographical origin of the strains

We checked whether there are differences in the transcriptomic response to cold depending on the origin of the strains in the two different *Drosophila* species. To do that, we used RNA-seq from ovaries of females after a cold treatment (see Methods).

PCA analysis on normalized gene counts revealed that PC1 (42% of the variance) separates control samples from those exposed to cold only for the strains dmgoth63 and dmsj23 (Fig. S5A, S6B). These strains, although being from distinct geographical origins, were the most sensitive to cold stress among the four strains studied (Fig. S1B). To further investigate whether differences in transcriptomic responses to cold associate with variations in CCRT among strains, we conducted a Mantel test. We obtained a Mantel statistic of *r* = 0.486 with a p-value of 0.167. Although the correlation was not statistically significant, likely due to the limited number of strains, it also suggests a moderate association between transcriptional and phenotypic responses to cold. In further detail, in *D. melanogaster*, the transcriptomic response to cold showed an increased number of DEGs compared to the starvation response (DEGs ranging from 414 to 1,060) (Fig. 2A). The response to cold showed a tendency for up-regulation, except for dmgoth101 (Fig. 2A), and it was strain-specific, showing many DEGs in only one of the strains tested (51% (555) of the up-regulated and 79% (681) of the down-regulated genes were found in a strain-specific manner) (Fig. S8C). Notably, dmgoth63 and dmsj23, exhibiting increased CCRT, shared the most DEGs (258 up-regulated and 48 down-regulated genes) (Fig. S8C and Fig. S1B). We also found a significant overlap between the DEGs and previous transcriptomic analysis after cold stress, suggesting that the observed response was indeed triggered by cold stress (Fig. S7B) [81]. GO analyses revealed that dmgoth63 and dmsj23 (more sensitive strains) have proteolysis and lipid metabolism/catabolism as common biological processes in the up-regulated DEGs, whereas down-regulated genes were mostly involved with fatty acid elongation process (Table S5). Interestingly, dmgoth101 and dmsj7 (more resistant strains) also had common enriched processes, for which up-regulated genes were enriched in functions like innate immune response, transmembrane transport, and antibacterial response; whereas common down-regulated genes were associated with vitelline membrane formation and chorion containing eggshell formation (Table S5).

On the other hand, in *D. simulans*, no clear sample clusters were observed in the PCA analysis based on geographical origin or phenotypic response (Fig. S5C and S6D). Moreover, Mantel tests suggested no association between transcriptional and phenotypic responses to cold (*r* = 0.086 with a p-value of 0.333). In this species, we also found more DEGs in response to cold compared to starvation conditions, except for dssj9 (Fig. 2B). The number of DEGs in this species ranged from 1 to 779, also showing an overall increase of up-regulated genes (Fig. 2B). Although the transcriptomic response to cold seems to be strain-specific (69% (807) of up-regulated and 96% (201) of down-regulated genes were found in a single strain), dsgoth31 and dssj27 were the two strains that shared more DEGs (225 up-regulated and 7 down-regulated genes) (Fig. S8D). In fact, these two strains had the lowest CCRT values among all the strains tested (Fig. S1B). Finally, GO analysis also revealed strain-specific patterns of gene expression changes upon cold (Table S5).

Overall, we found that the transcriptomic response upon cold stress was not likely to be linked with the geographical origin of the populations in any of the species studied.

### TEs are not enriched near differentially expressed genes

TEs have been shown to modulate the expression of genes under stress conditions in different organisms due to their ability to distribute stress-response elements throughout the genome that can be activated during stress, and reprogram gene networks, or by modifying the epigenetic regulation of a locus [40]. Thus, we wanted to test whether TE insertions are enriched in the promoter region of the previously observed DEGs. To do that, we performed permutation tests by intersecting TE positions with promoter regions using the R package *regioneR* (Table 1 and Fig. S9 and S10) [72].

**Table 1:** Analysis of TE insertion enrichment near DEGs

	Strain	Starvation		Cold	
		Up	Down	Up	Down
*D. melanogaster*	dmgoth101	**0.005** ↑	**0.001**↓	0.193	**0.039** ↓
	dmgoth63	0.371	0.763	0.862	0.084
	dmsj23	0.150	0.741	**0.049** ↓	0.174
	dmsj7	0.729	0.092	**0.002** ↓	0.099
*D. simulans*	dsgoth31	0.884	0.681	**0.037** ↑	0.424
	dsgoth613	0.751	0.562	0.205	0.272
	dssj27	0.127	0.376	**0.012** ↓	0.073
	dssj9	0.160	0.376	0.914	-

Statistically significant values resulting from the permutation tests (see Methods) are highlighted in bold. Arrows indicate enrichment (up) or depletion (down) of TEs near DEGs. Strains were named based on their location of origin: temperate strains are named “goth” (from Gotheron, France), while tropical strains are named with “sj” (from São José do Rio Preto, Brazil)

Under starvation conditions, we observed that TE insertions were neither enriched nor depleted near up- or down-regulated genes (Table 1 and Fig. S9). The only exception was observed in the temperate *D. melanogaster* strain dmgoth101, where TE insertions were enriched near up-regulated genes and depleted near down-regulated genes (Table 1 and Fig. S9). However, after cold treatment, we found that up-regulated genes were depleted of TE insertions in the two *D. melanogaster* tropical strains and in one of the two *D. simulans* strains from the same origin (Table 1 and Fig. S10). Additionally, we observed a depletion of TE insertions near the down-regulated genes in the *D. melanogaster* temperate strain dmgoth101, and an enrichment near the up-regulated genes in the *D. simulans* temperate strain dsgoth31 (Table 1 and Fig. S10).

Our findings suggest a lack of general enrichment of TE insertions near DEGs under any of the stress conditions in both species.

### Starvation or cold stress does not appear to cause a general activation of TEs

Although it is not a general rule for all TEs, they have been reported to be activated under stress conditions, meaning that stress can induce their expression and transposition [40]. This stress-induced TE activation has often been involved in adaptation, because it can lead to an increased mutation rate, generating variability upon which natural selection can act [40]. However, the relationship between TE activation and stress is complex. TEs may be either activated or repressed under stress conditions, and the extent of their expression changes seems to be stress-specific [38, 83, 84]. To know whether TEs are activated or repressed under starvation or cold conditions, we used the same RNA-seq data from ovaries to measure TE expression at the family level in all the strains and species (see Methods).

PCA analysis showed that TE expression was strain-specific (Fig. S4B and S4D), with control samples clustering with stressed samples in both *D. melanogaster* and *D. simulans* (Fig. S5B, S5D and Fig. S6B, D). Indeed, in starvation conditions, only some strains had differentially expressed TE families (Fig. 2C-D and Fig. S11). In *D. melanogaster*, strain dmgoth101 had 13 up-regulated TE families, while only one TE family was down-regulated in dmsj7 (Fig. 2C and Fig. S11). In *D. simulans*, dssj27 and dssj9 each showed an up-regulation of three TE families, whereas dsgoth31 and dsssj27 had two and seven down-regulated TE families, respectively (Fig. 2D and Fig. S11).

After the cold treatment, in *D. melanogaster*, we found strains with none or just two TE families up-regulated (dmgoth63, dmsj23, and dmsj7), whereas 14 TE families were activated in dmgoth101 (Fig. 2C and Fig. S11). Moreover, between one and three TE families were down-regulated in all the strains except in dmgoth63, in which we observed 13 TE down-regulated families (Fig. 2C and Fig. S11). In *D. simulans*, the number of activated TE families upon cold stress was also low, ranging from zero in dssj9 to six in dsgoth613 (Fig. 2D and Fig. S11). TE family decrease in expression was observed only in two strains: dsgoth613 showed one TE family down-regulated, whereas dssj27 showed 24 (Fig. 2D and Fig. S11).

Finally, in *D. melanogaster*, a positive correlation between TE RNA counts and piRNA counts was previously described in wild-type strains in control conditions [61, 68]. However, the standard normalization method relying on miRNAs does not allow to detect a significant correlation between TE RNA variation and TE piRNA variation cold stress in any of the strains (all p-values > 0.05, Fig. S12).

In summary, we found that neither starvation nor cold stress induced a general TE activation in the strains tested, also suggesting a strain-specific regulatory response.

### Cold stress is associated with chromatin changes in TE flanking regions

Temperature can affect chromatin conformation, and in turn modulate gene and TE regulation [85–87]. Hence, we investigated how cold can affect chromatin conformation and whether the observed mild TE expression changes in cold stress are due to changes in chromatin accessibility. To do that, we performed chromatin immunoprecipitation sequencing (ChIP-seq) (see Methods) focusing on two histone marks: H3K4me3 (Histone 3 Lysine 4 trimethylation), which is associated with active and canonical transcription; and H3K9me3 (Histone 3 Lysine 9 trimethylation) which is generally considered as a silencing chromatin mark and is also associated with the production of TE-derived silencing piRNAs [88].

As expected, we found that transcription start sites (TSSs) were globally enriched in H3K4me3 and depleted in H3K9me3 in all samples (Fig. S13). We then investigated H3K4me3 and H3K9me3 profiles in the 2 kb flanking regions of all TEs in *D. melanogaster* and *D. simulans* strains (see Methods). We found an overall enrichment of the repressive mark H3K9me3, whereas there was an overall depletion of the open-chromatin mark H3K4me3 in the TE flanking regions (Fig. S14). This result is associated with the previously described pattern of TE silencing through epigenetic mechanisms [88, 89]. Moreover, all tested strains showed significant differences in histone enrichment between control and cold conditions (Wilcoxon signed-rank test, *p* < 0.05), except for dmj23 with H3K4me3. This result might suggest an overall chromatin modulation at a TE copy level in both species in cold stress.

**Fig. 3:**
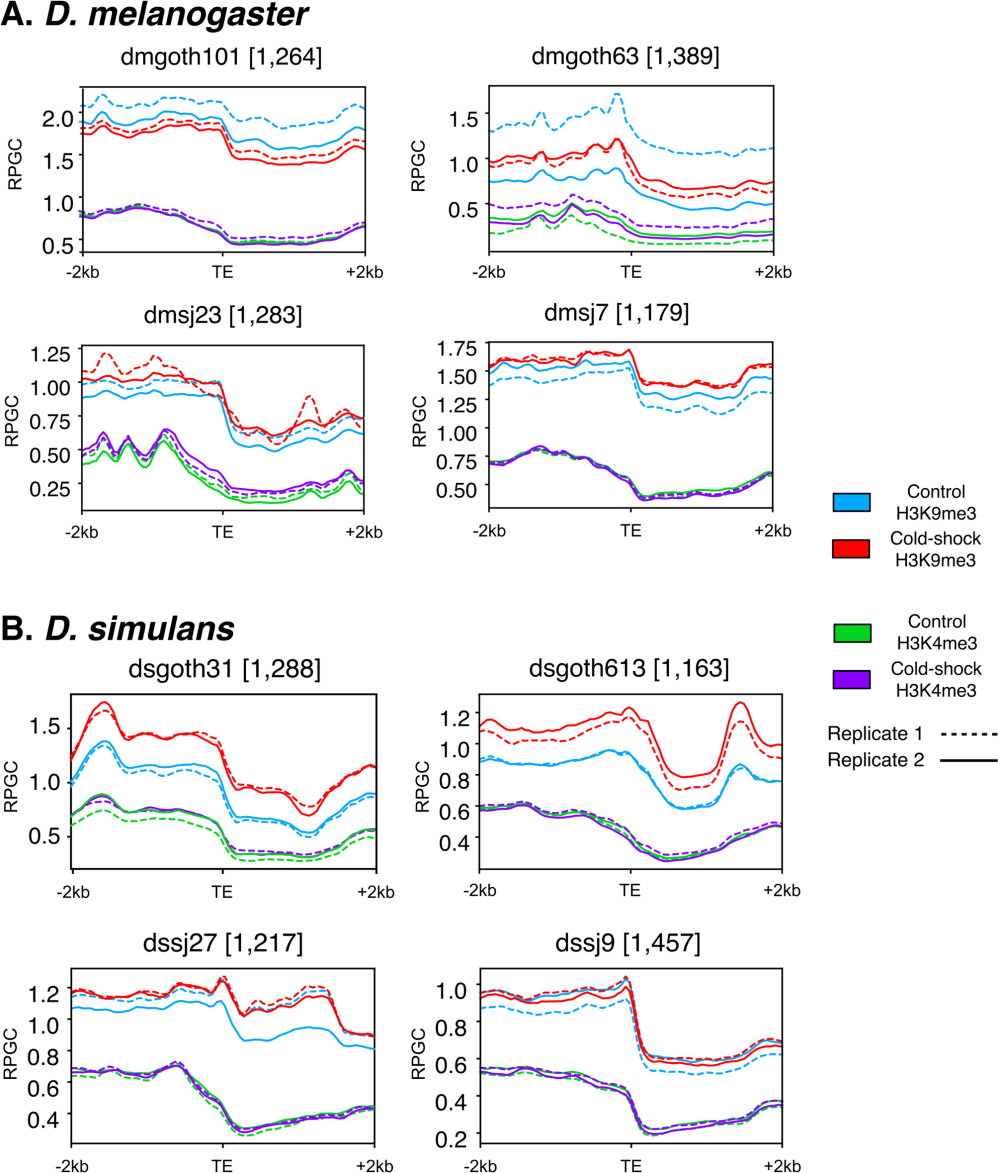
Cold stress is associated with global changes in TE chromatin accessibility in full-length TEs. H3K4me3 and H3K9me3 profiling on 2 kb flanking regions of full-length TE insertions in **A**) *D. melanogaster* and **B**) *D. simulans*. Strains were named based on their location of origin: temperate strains are named “goth” (from Gotheron, France), while tropical strains are named with “sj” (from São José do Rio Preto, Brazil). Numbers in brackets represents the total of full-length TE insertions observed in each strain

We then focused on full-length (FL) insertions due to their potential mutagenic effects (see Methods) [36]. We identified 1,278 and 1,288 FL copies on average in all *D. melanogaster* and *D. simulans* strains, respectively (Table S6). In both species, we found similar results using FL copies as for all TEs: enrichment of H3K9me3 and depletion of H3K4me3 (Fig. 3). Moreover, we observed in both species a few FL insertions with an enrichment of H3K4me3 in their flanking upstream region, more specifically in DNA elements, whereas LINEs and LTRs are depleted (Fig. 3, S15 and S16).

In summary, although we found a weak transcriptional response of TEs upon stress, we could detect changes in chromatin accessibility in their flanking regions.

### The amounts of TE-chimeric transcripts are not affected by stress

**Fig. 4:**
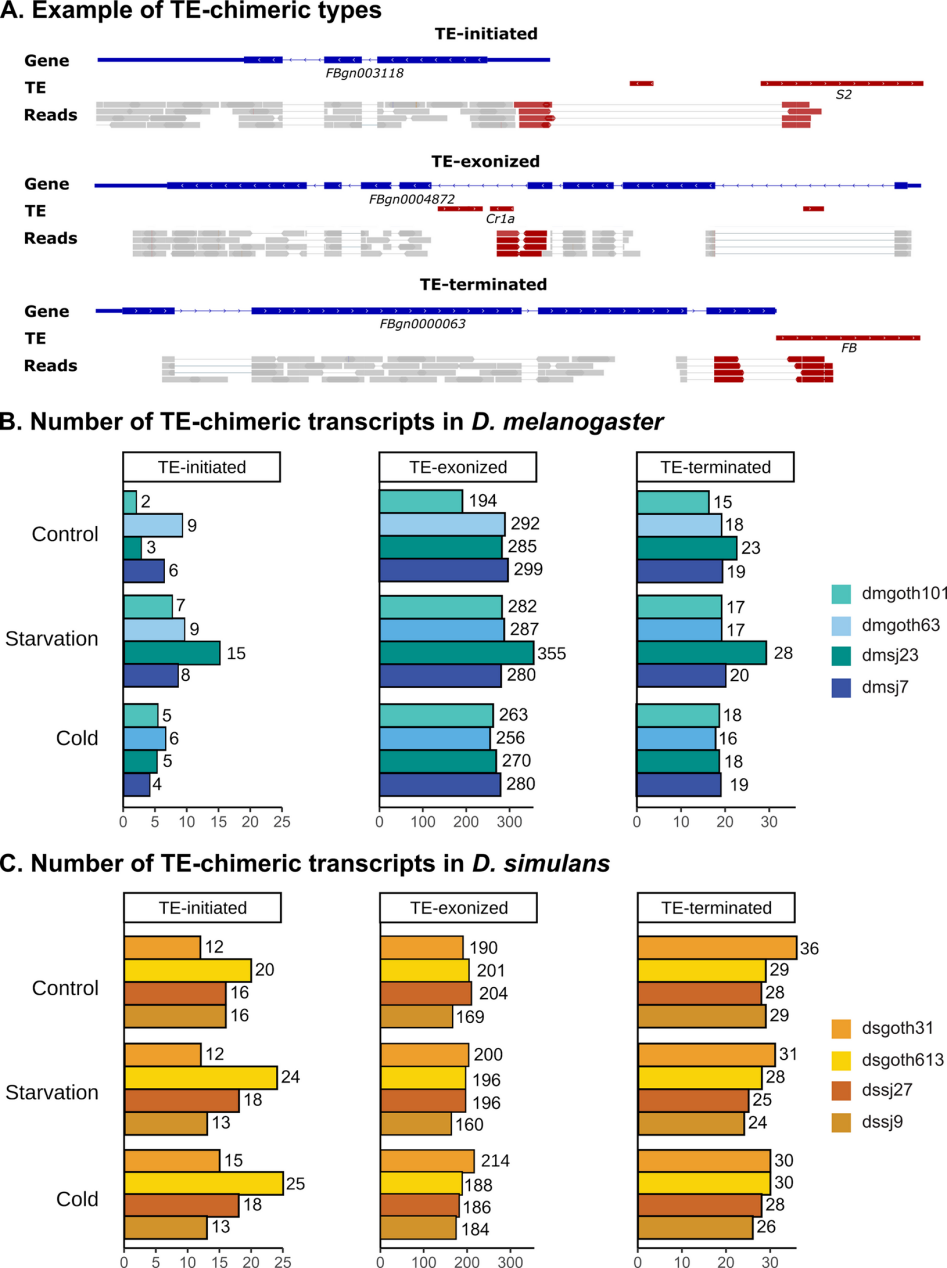
Types and number of chimeric transcripts generated in each strain. **A**) Schematic representation illustrating each type of chimeric transcript: TE-initiated, TE-exonized, and TE-terminated. TE-initiated transcripts are chimeric transcripts with a TE transcription start site, TE-exonized transcripts have TE sequences incorporated into the transcript either partially or as full-length exons; and TE-terminated transcripts have a TE transcription termination site. Genes are shown in blue, TEs and paired-end reads mapping to TEs are shown in red, and all other paired-end reads are shown in grey. Arrows represent the direction of the gene/transcript. For clarity and due to space limitations, a reduced coverage depth of four reads over the genes is shown. **B**) Number of chimeric transcripts found in *D. melanogaster* strains and in **C**) *D. simulans* strains under the different experimental conditions and classified according to the chimeric transcript type. Strains were named based on their location of origin: temperate strains are named “goth” (from Gotheron, France), while tropical strains are named with “sj” (from São José do Rio Preto, Brazil)

TEs can also contribute to the complexity and diversity of the transcriptome by the generation of gene-TE chimeric transcripts [55–57, 90]. Although the generation of gene-TE chimeric transcripts has been recently studied in the same *D. melanogaster* natural populations used in this work [56], their contribution to transcript diversification has not yet been studied in stress conditions transcriptome-wide, neither in *D. melanogaster* nor in *D. simulans*. Therefore, we used the pipeline *ChimeraTE* to detect different types of TE-chimeric transcripts in *D. melanogaster* and *D. simulans* flies exposed to starvation and cold stress (Fig. 4A) [56].

In *D. melanogaster*, an average of 291 TE-chimeric transcripts were previously identified in the same strains analysed in this study under control conditions [56]. We then checked whether stress could change the number of TE-chimeric transcripts compared to the control. In starvation conditions, we found an average of 331 TE-chimeric transcripts, whereas in cold we found 290 (Fig. 4B). Hence, there was not a significant difference in the number of TE-chimeric transcripts generated when comparing control with any of the stress conditions (ANOVA, *p* = 0.296 in starvation and *p* = 0.964 in cold, respectively).

Regarding *D. simulans*, we found an average of 252 TE-chimeric transcripts in control condition, 248 in starvation, and 254 in cold stress (Fig. 4C). Overall, we observed a decreased number of TE-chimeric transcripts compared to *D. melanogaster*, as expected considering the lower TE/gene content in this species [91]. Again, we did not find significant differences in the number of TE-chimeric transcripts when comparing control with stress conditions (ANOVA, *p* = 0.721 in starvation and *p* = 0.877 in cold, respectively).

Then, we investigated if the generated TE-chimeric transcripts were shared between conditions. We found that the vast majority of them were found in all the experimental conditions, suggesting that stress does not trigger a strong TE-chimeric transcript production (Fig. S17).

In both species and in all the conditions, we found a positive correlation between the number of TE-chimeric transcripts and the copy number of TEs (Fig. S18). The *roo* family has the highest frequency of TE-chimeric transcripts in both species and conditions, as it has been demonstrated in *D. melanogaster* [56, 57] (Fig. S18).

In summary, these results indicate that in general, stress does not increase the generation of TE-chimeric transcripts in any of the species.

### TE-chimeric transcripts in *Paris*, *Sulf1*, and *GD24271* genes are associated with increased transcript levels upon stress

Even though stress does not appear to strongly induce the production of TE-chimeric transcripts, we tested whether those that are generated might be related with changes in nearby gene expression by identifying DEGs with TE-chimeric transcripts in response to stress.

**Fig. 5:**
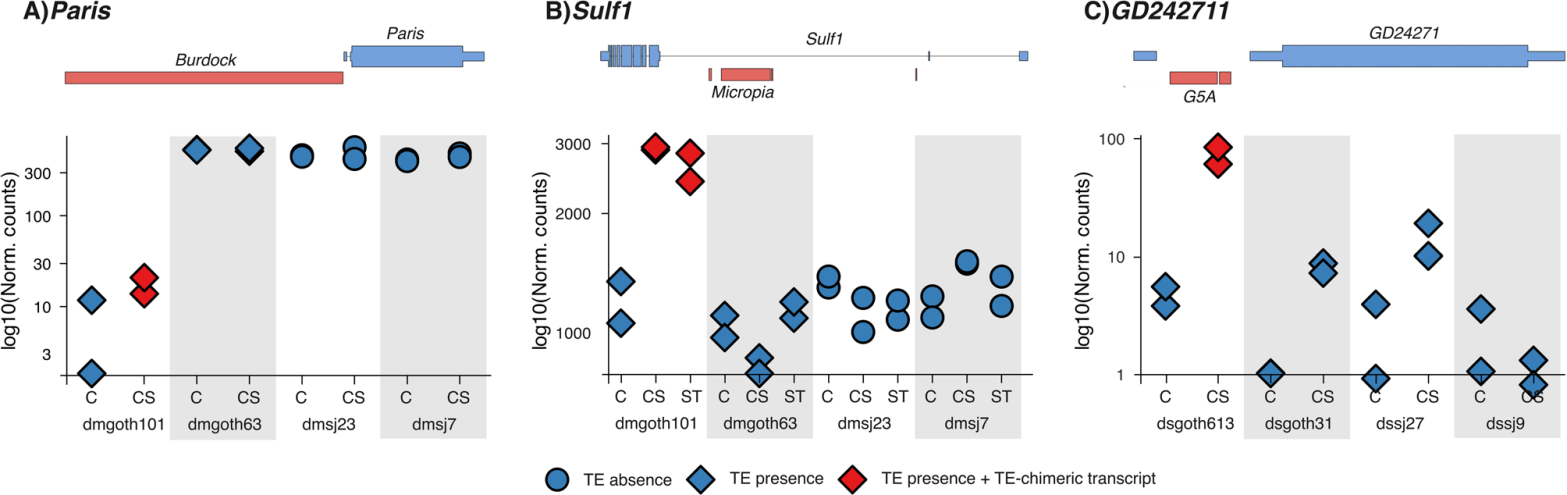
DEGs generating chimeric transcripts. **A**) Schematic representation of the *D. melanogaster Paris* gene, with a *Burdock* insertion located upstream in dmgoth101 and dmgoth63 strains, and absent in dmsj23 and dmsj7. The TE-chimeric transcript is observed in dmgoth101, only in cold stress conditions, where the *Paris* gene is up-regulated. **B**) Schematic representation of the *D. melanogaster Sulf1 *gene, which has a *micropia* insertion located in the intron in the dmgoth101 and dmgoth63 strains. In dmgoth101, it generates a TE-chimeric transcript in starvation and cold stress conditions, where the *Sulf1* gene was up-regulated. **C**) Schematic representation of the *GD24271* gene in *D. simulans*, which has a *G5A* insertion in all strains, but it produces a chimera only in dsgoth613 in cold conditions, in which the gene was up-regulated. C: control conditions; CS: cold stress conditions; ST: starvation conditions

We found three TE-chimeric transcripts in the DEGs *Paris* and *Sulf1* in *D. melanogaster*, and *GD24271* in *D. simulans*, respectively. The upstream region of the *Paris* gene, a transcriptional repressor involved in the regulation of mitochondrial biogenesis, has a polymorphic *Burdock* insertion only present in the *D. melanogaster* temperate strains. This TE insertion generates a TE-chimeric transcript in the *Paris* gene only in dmgoth101, for which we observed up-regulation upon cold stress (Fig. 5). Furthermore, the gene *Sulf1* in *D. melanogaster* has a polymorphic *Micropia* element located in its intron only in the temperate strains. *Sulf1* encodes a heparan sulfate modifying enzyme, regulating the *Wg* signaling, essential during embryogenesis and adult homeostasis. This gene produces a TE-chimeric transcript in dmgoth101, where we found up-regulation upon both stresses (Fig. 5). Finally, a *G5A* element located upstream of the *GD24271* gene in *D. simulans* of both temperate and tropical populations generates a TE-chimeric transcript only in the temperate strain dsgoth613 after cold stress, where the gene was found up-regulated (Fig. 5). The ortholog gene in *D. melanogaster* is predicted to be involved in protein targeting to the membrane.

In all three cases, increased gene expression was observed only in strains producing TE-chimeric transcripts, suggesting that chimera formation may be linked to elevated gene expression.

## Discussion

In our phenotypic assays, natural variability in response to starvation and cold stress was observed in both *D. melanogaster* and *D. simulans*. For both traits and species, the phenotypic response was sex- and strain-specific. In *D. melanogaster*, no differences in starvation resistance were found between flies from temperate (France) and tropical (Brazil) origins. However, in *D. simulans*, temperate flies exhibited greater resistance than their tropical counterparts. Clinal patterns of starvation resistance were previously described in *D. melanogaster* from Indian and Australian populations, being starvation resistance negatively correlated with latitude [26, 28, 92]. However, this pattern of clinal variation in starvation was not observed in South and North American populations [26, 31, 93], suggesting inconsistent selection on starvation across continents [29]. The lack of correlation between phenotypic response to starvation and the population of origin in *D. melanogaster* may be due to high variability between strains within a population. Conversely, our results for *D. simulans* do not align with previous works showing a weak negative correlation between resistance to starvation and latitude in female flies from the Australian coast [30]. These findings suggest diverse patterns in starvation resistance between populations, similar to *D. melanogaster*. Concerning cold stress, significant differences between temperate and tropical populations were found in both *D. melanogaster* and *D. simulans*, consistent with the literature [28, 30, 93]. Populations from temperate regions experience harsh winters with freezing temperatures, which exert a high selective pressure for increased cold tolerance.

The originality of the present work is the analysis of the phenotypic response to stress in light with transcriptome changes. We found contrasting patterns of geographical differentiation across species. In *D. melanogaster*, the transcriptomic response to stress was conserved between geographical origins. Strains that were more resistant to starvation exhibited a more robust transcriptomic response to the stress. On the contrary, in *D. simulans,* we observed population-specific patterns, although with more DEGs in tropical strains, in line with their lower resistance compared to temperate strains.

After cold stress, several studies suggested that there is a large number of genes exhibiting geographically-dependent expression plasticity in *D. melanogaster* [86, 87, 94, 95]. In this species, although temperate strains are the ones with a higher number of DEGs, we found that transcript abundances showed less variation in strains with an increased resistance to cold. Gene canalization, the phenomenon in which gene expression remains relatively stable despite environmental variations, has been described to be important in the response to cold in temperate populations of *D. melanogaster* [86, 87, 95]. In that sense, it is hypothesized that the canalization of the expression changes in temperate populations might be responsible for the increased tolerance to cold [95, 96]. In our results, more resistant phenotypes might result from a stronger canalization process, since their transcriptomic profile does not change a lot in response to the stress.

Some TE insertions have previously been shown to affect nearby gene expression in response to stress in *D. melanogaste*r [43, 44, 97]. Examples of that can be found in oxidative stress, desiccation, and copper tolerance [13, 14, 45, 84, 98]. Indeed, TE insertions were found to be depleted nearby DEGs in oxidative stress and desiccation. This is consistent with the fact that we did not find a general enrichment of TE insertions nearby DEGs in *D. melanogaster* or *D. simulans* neither in starvation nor in cold stress. In fact, we found more cases of depletion than enrichment. However, we cannot discard that some specific insertions could be responsible for the differences in gene expression detected in stress conditions.

TE families can be stress-activated, depending on stress type and TE family [40, 99]. In our study, neither starvation nor cold induced a general TE activation in both *D. melanogaster* and *D. simulans*. However, more TE expression changes occurred after cold stress than starvation in both species. These results are consistent with previous studies showing that although stress activates TEs, this is not a generalized activation process, and for some families the stress induces repression [45–49, 84]. Moreover, our findings indicate that TE responses to stress are highly dynamic and context-dependent, varying both across genotypes and between species. This variability suggests that TE regulation under stress may represent a labile trait, influenced by genetic background and environmental context. In some cases, such plasticity could facilitate rapid genomic responses to environmental challenges, potentially fueling phenotypic innovation and adaptive evolution across generations [40].

So far, most of the genome-wide analyses of TE expression have been focused at the family level. Despite the emergence of some methods using short-read sequencing datasets to develop copy-level analysis [100, 101], assigning short-reads to specific TE copies is still not trivial [90]. The availability of new long-read RNA-seq technologies would make it possible to identify and quantify TE expression at the copy-level [102], thus achieving an essential and broader understanding of the impact of TE expression in the genome.

TEs can also contribute to the complexity and diversity of the transcriptome by the generation of gene-TE chimeric transcripts. Recently, ~ 1.12% of the genes were found to have TE-chimeric transcripts in the same *D. melanogaster* strains analysed in the present study [56]. However, the generation of these TE-chimeric transcripts have been shown to vary between strains as well as body parts [57, 58]. Indeed, TEs were found to contribute ~ 9% to the global *D. melanogaster* transcriptome and ~ 19% to the body part-specific transcriptome across five different natural populations [57]. Here, we checked for the first time the generation of TE-chimeric transcripts in *D. simulans*, finding an average of 1.49% genes generating this type of transcripts. Moreover, we found that the generation of these TE-chimeric transcripts does not change after starvation or cold stress in any of the species tested. However, we found three putative candidate TEs generating TE-chimeric transcripts that might be inducing gene expression changes after stress in temperate strains: two in *D. melanogaster* and one in *D. simulans*. Although the function of these genes has not been described as being associated with stress, further experiments are needed to validate the presence of the TE-chimeric transcript with gene expression changes and the phenotypic response.

Laboratory-based stress experiments are challenging, and it is difficult to determine if the stress applied is biologically relevant. In our tested conditions, TEs had minimal impact on stress-induced transcriptional responses. However, we cannot discard the possibility that a stronger, more detrimental and realistic stress might yield different TE impacts. In addition, our approach did not allow us to identify particular TE insertions with important effects on gene expression changes.

In summary, this study highlights the importance of analysing the transcriptomic variability between different populations and in different species to better understand their phenotypic responses to stress.

## Conclusions

Our study reveals substantial natural variation in the phenotypic and transcriptomic responses to environmental stress across strains and species of *Drosophila*. While both *D. melanogaster* and *D. simulans* displayed strain- and sex-specific responses to starvation and cold, only *D. simulans* showed an association between geographic origin and starvation resistance. Furthermore, transcriptomic analyses showed contrasting patterns of stress response between species: *D. melanogaster* exhibited more conserved and potentially canalized gene expression changes, particularly in cold-resistant strains, whereas *D. simulans* showed more population-specific responses. In this study, TEs had a limited genome-wide impact on stress-induced expression changes, with no evidence for generalized TE activation or enrichment near DEGs. However, we identified a few TE-chimeric transcripts that may influence gene expression in response to stress.

## Supplementary Information

Below is the link to the electronic supplementary material.

Supplementary Material 1

Supplementary Material 2

Supplementary Material 3

Supplementary Material 4

Supplementary Material 5

Supplementary Material 6

Supplementary Material 7

Supplementary Material 8

## Data Availability

The datasets generated and/or analysed during the current study are available in the published article (and its supplementary information files). Raw sequence reads are deposited in the SRA (BioProject PRJNA1029344).
