# Supplementary material for: Gene and transposable element expression in response to stress in temperate and tropical populations of Drosophila
Source: Mob DNA. 2025 Sep 30;16:35. doi: 10.1186/s13100-025-00372-x (PMC12487357; doi:10.1186/s13100-025-00372-x)

**SUPPLEMENTARY INFORMATION**

**Figure S1.** Natural variability to starvation and CCRT in *D. melanogaster* and *D. simulans*. **A)** Barplot showing for each of the tested strains (x axis), the mean lifespan in hours under starvation conditions for both *D. melanogaster* and *D. simulans* males (left) and females (right). Each bar represents the average calculated from 15 biological replicates (± s.d. of the mean). **B)** Violin plot showing for each of the tested strains (x axis), the mean CCRT in minutes for both *D. melanogaster* and *D. simulans* males (left) and females (right). Each violin plot represents the value of the 48 individuals tested. Fly strains selected for downstream analysis are highlighted in bold. Strains were named based on their location of origin: temperate strains are named “goth” (from Gotheron, France), while tropical strains are named with “sj” (from São José do Rio Preto, Brazil).


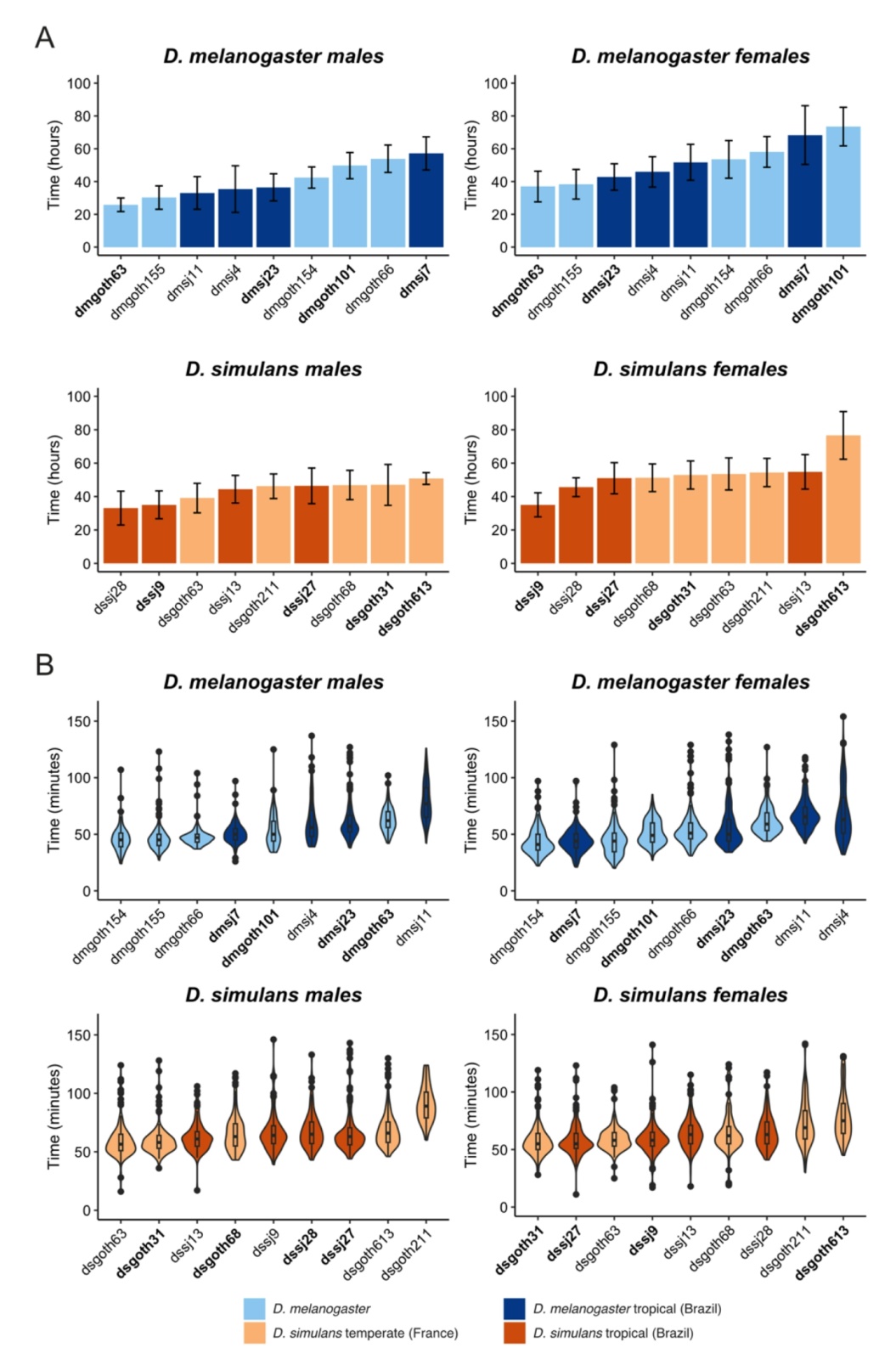



**Figure S2.** Kaplan–Meier survivorship curves for *D. melanogaster* and *D. simulans* under control conditions.


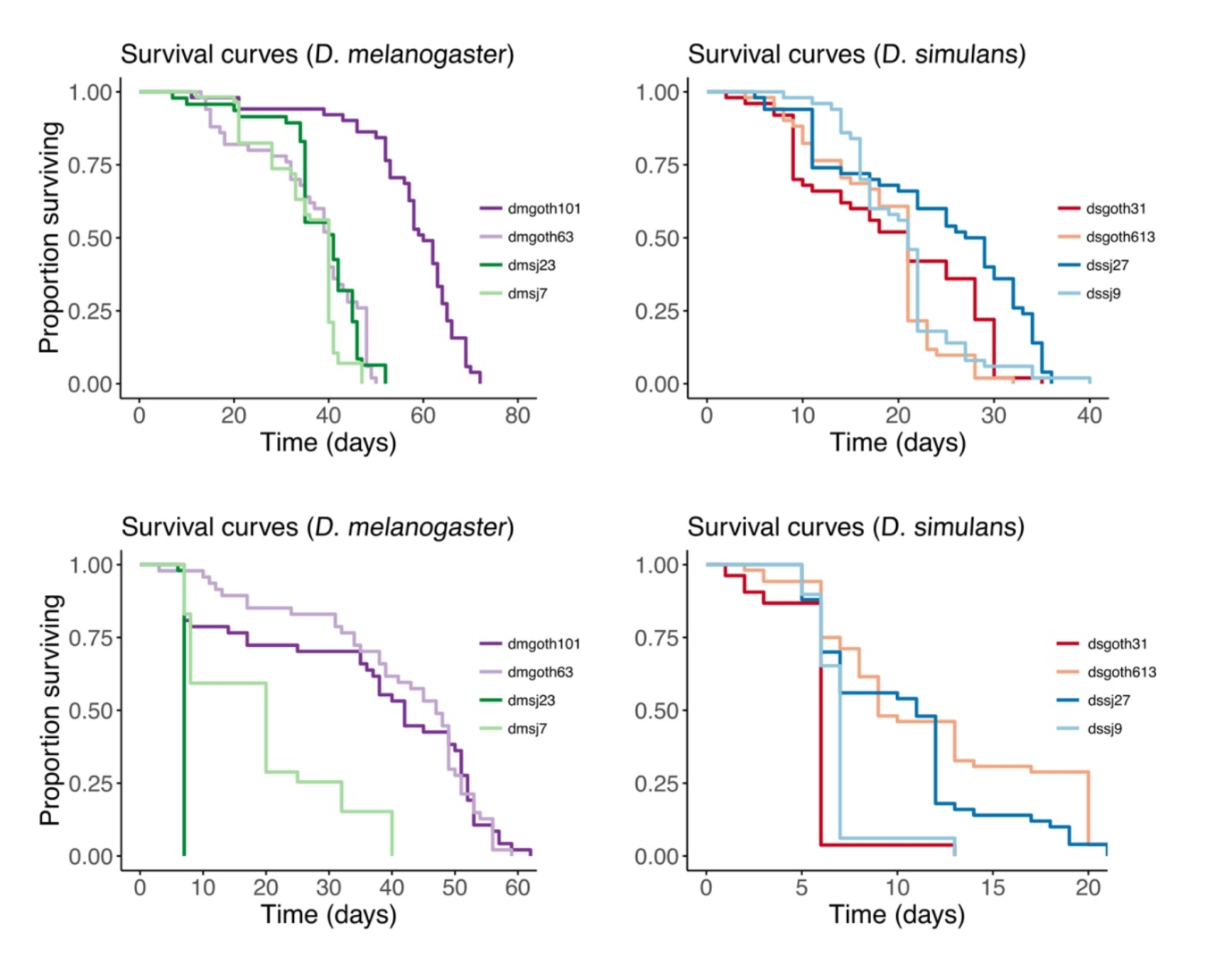



**Figure S3.** Histograms and density distributions of mean lifespan in starvation and mean CCRT in **A)** *D. melanogaster* and **B)** *D. simulans*. dm: *D. melanogaster*; ds: *D. simulans*.


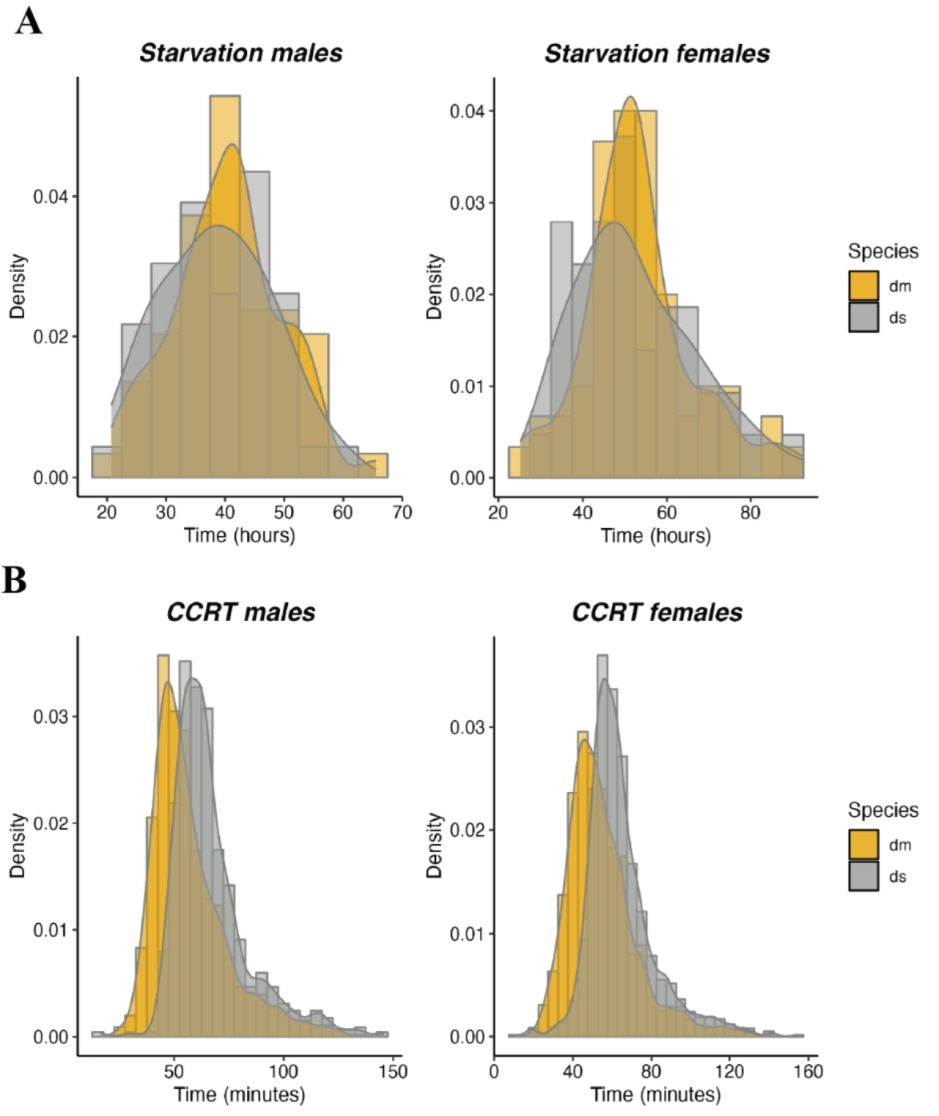



**Figure S4.** PCA plots in control conditions for **A)** *D. melanogaster* genes, **B)** *D. melanogaster* TEs, **C)** *D. simulans* genes and **D)** *D. simulans* TEs. PCA was performed on normalized genes (A and C) and TE expression (B and D).


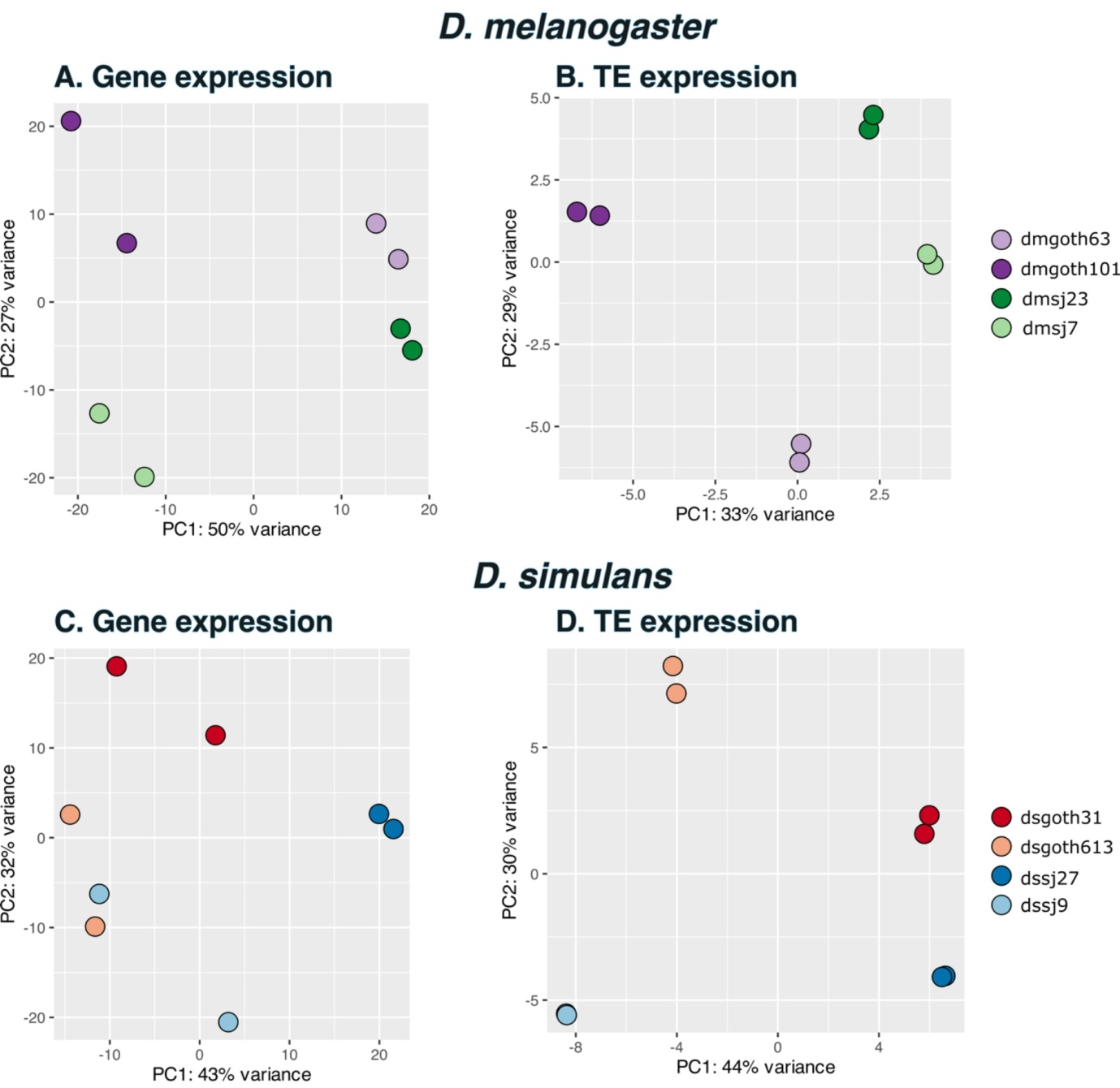



**Figure S5.** PCA plots in control and stress conditions (starvation and cold) for **A)** *D. melanogaster* genes, **B)** *D. melanogaster* TEs, **C)** *D. simulans* genes and **D)** *D. simulans* TEs. PCA was performed on normalized genes (A and C) and TE expression (B and D).


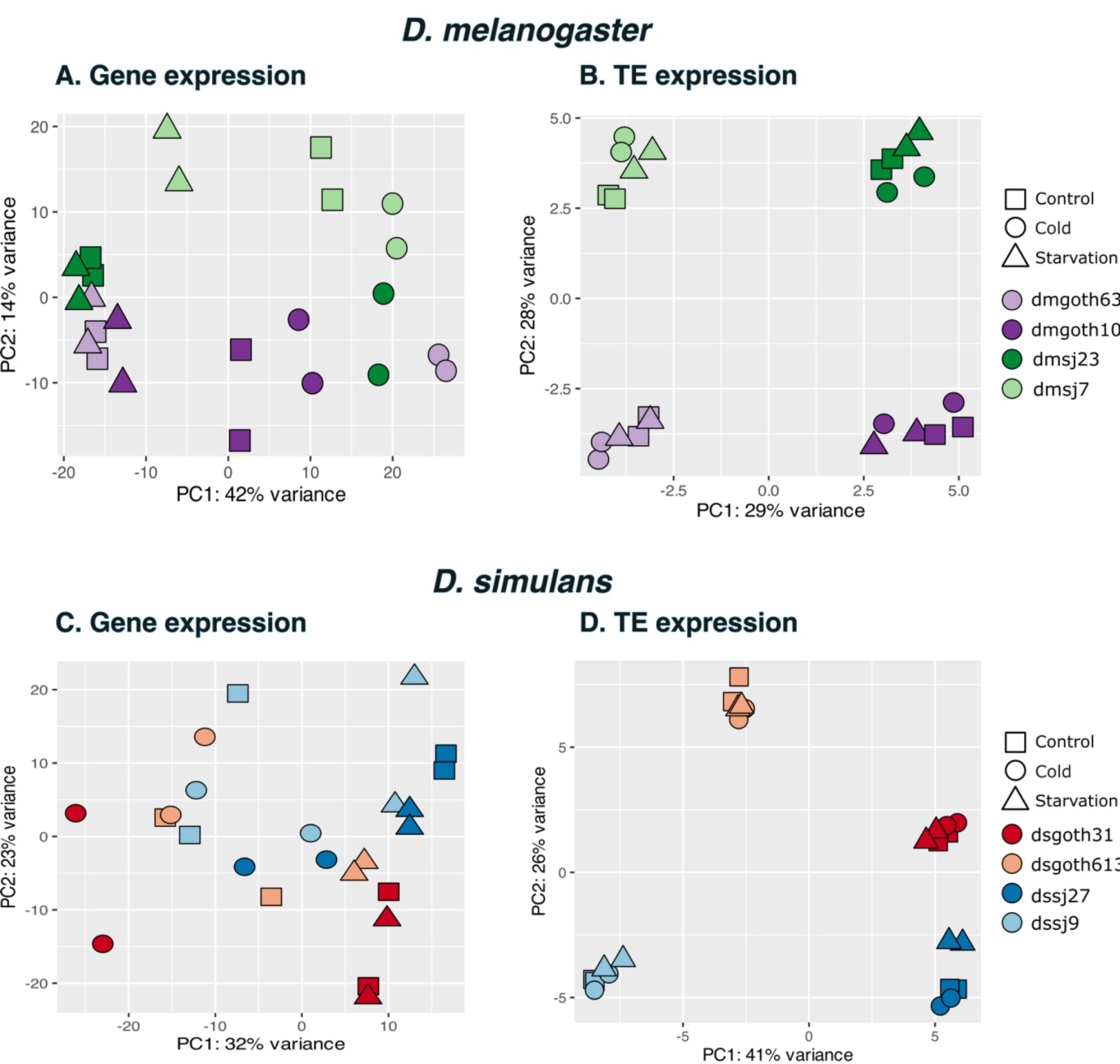



**Figure S6.** Heatmap of DEGs upon stress in at least one of the strains analyzed for **A)** *D. melanogaster* genes in starvation, **B)** *D. melanogaster* genes in cold stress, **C)** *D. simulans* genes in starvation and **D)** *D. simulans* genes in cold stress. Color legend represents the log2 normalized gene counts.


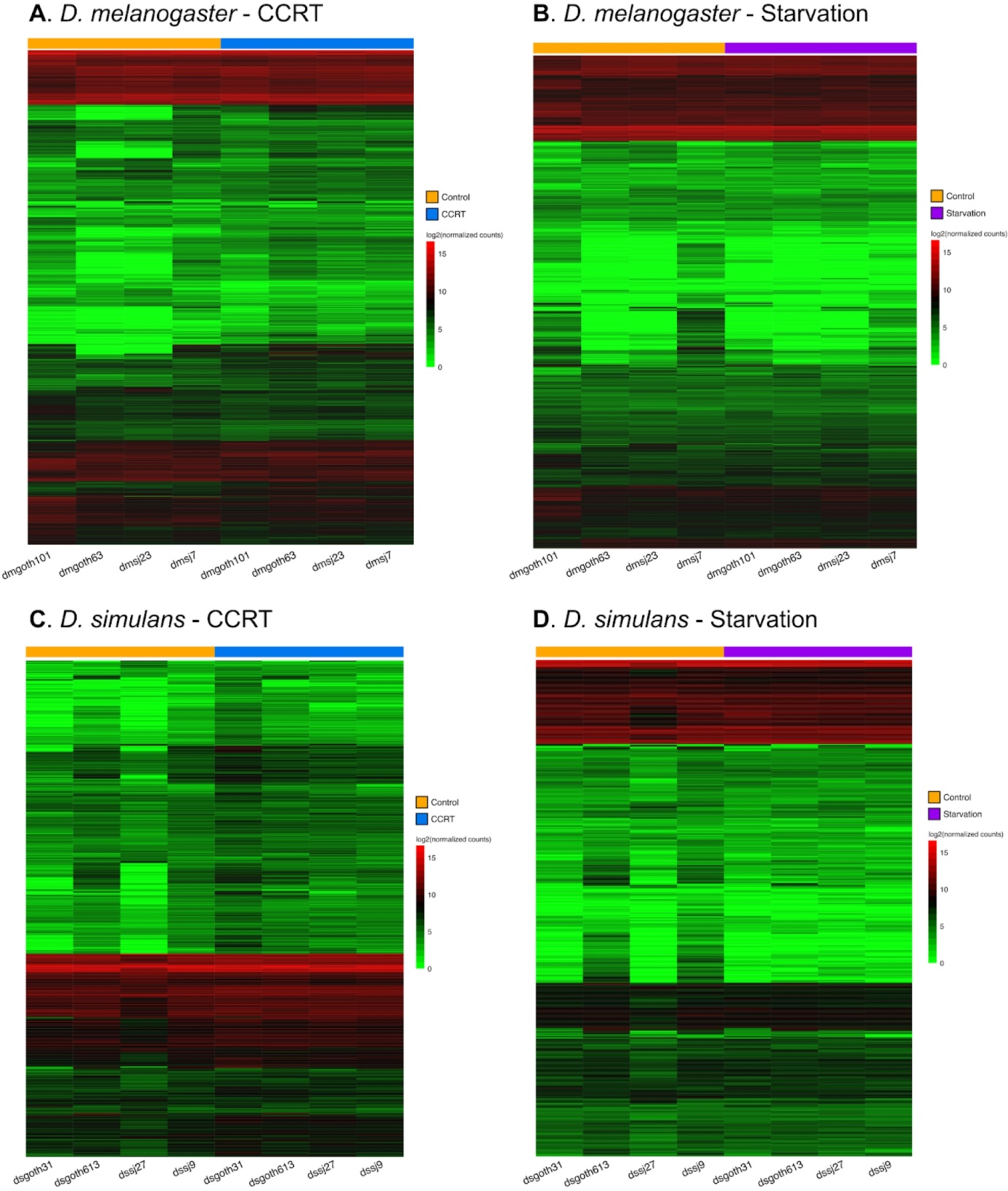



**Figure S7.** Enrichment of DEGs found in this work with previous transcriptomic analysis found in the literature for **A)** starvation and **B)** cold stress. *all* represent the overlap between the discovered DEGs and all the related candidate genes and *> 2* represent the overlap between the discovered DEGs and the subset of related candidate genes present in at least 2 independent studies. For each stress condition a common list of DEGs unifying the data obtained for all the strains using R. The previously available information that summarizes evidence from genome-wide association studies (GWAS), quantitative trait loci (QTL), gene expression, protein–protein interactions, and candidate gene studies to identify genes involved in starvation and cold stress was gathered (81). Finally, the R package *GeneOverlap* was used to test for each stress the overlap between the DEG list obtained in this work and the complete and subset list of candidate genes related.


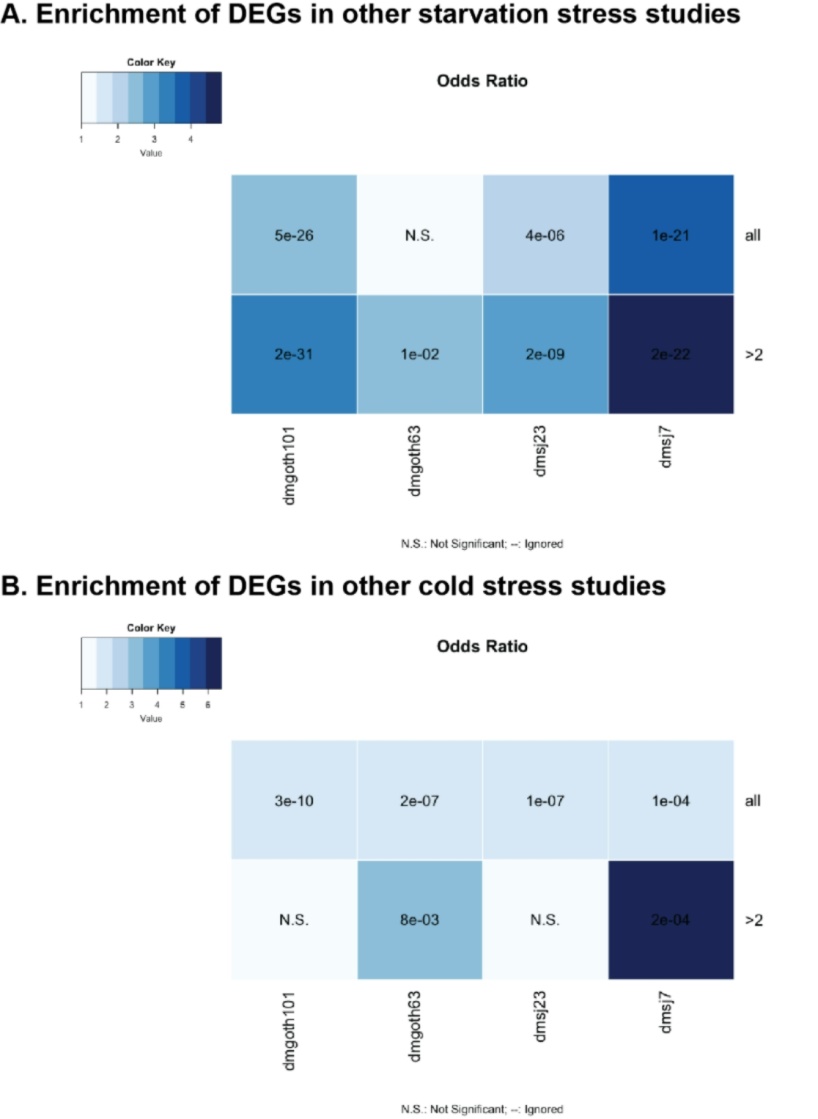



**Figure S8.** Upset graphs with shared DEGs between strains in starvation and cold conditions. **A)** Upset graph with the number of up-regulated (left panel) and down-regulated genes (right panel) shared between strains in response to starvation in *D. melanogaster* **B)** and in *D. simulans*. **C)** Upset graph with the number of up-regulated (left panel) and down-regulated genes (right panel) shared between strains in response to cold stress in *D. melanogaster* **D)** and in *D. simulans*.


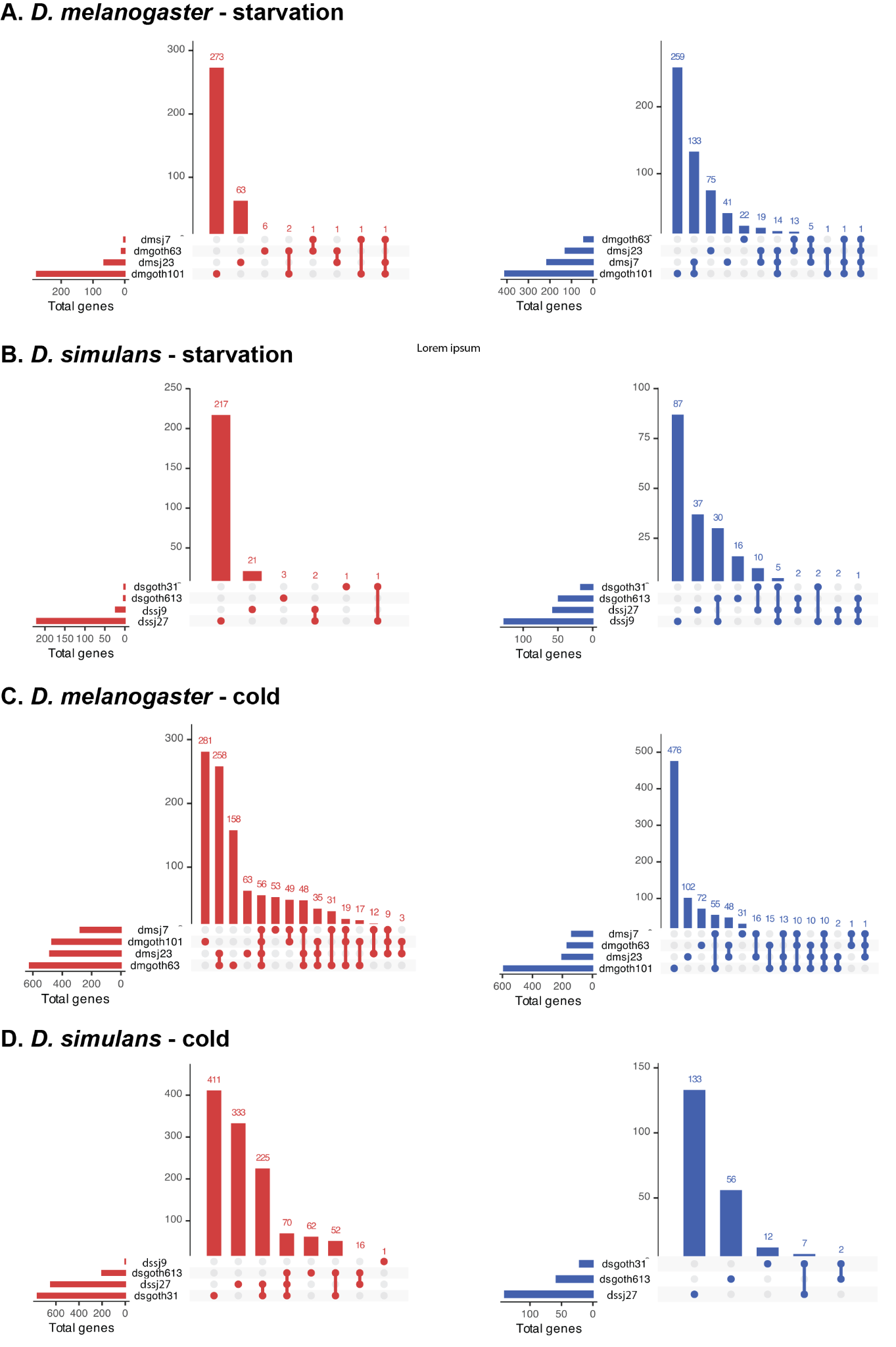


**Figure S9.** Results of the permutation test in all the strains tested after starvation in **A)** *D. melanogaster* and **B)** *D. simulans*. To test whether TEs are enriched in the promoter regions of DEGs, we firstly created *bed* files with the 3 kb upstream region for these genes. Then, we performed permutation tests by intersecting TE positions with promoter regions using the R package *regioneR* (72), parameter *randomization = resampleRegions*, with 1000 random samples. Therefore, for each set of up and down-regulated genes with size *n*, the package identifies how many of them have TEs at the 3 kb upstream region. Finally, it performs 1000 samples of *n* genes randomly, to test whether the observed number of genes with TEs is greater or lower than expected in a genome-wide comparison. Histograms show the frequency of genes with TEs in the promoter region after 1000 random sampling. Black lines represent the expected values, red lines represent type I error ɑ level, and green lines represent the observed values. Significant results are found when the black and green lines are in opposite directions regarding the red line.


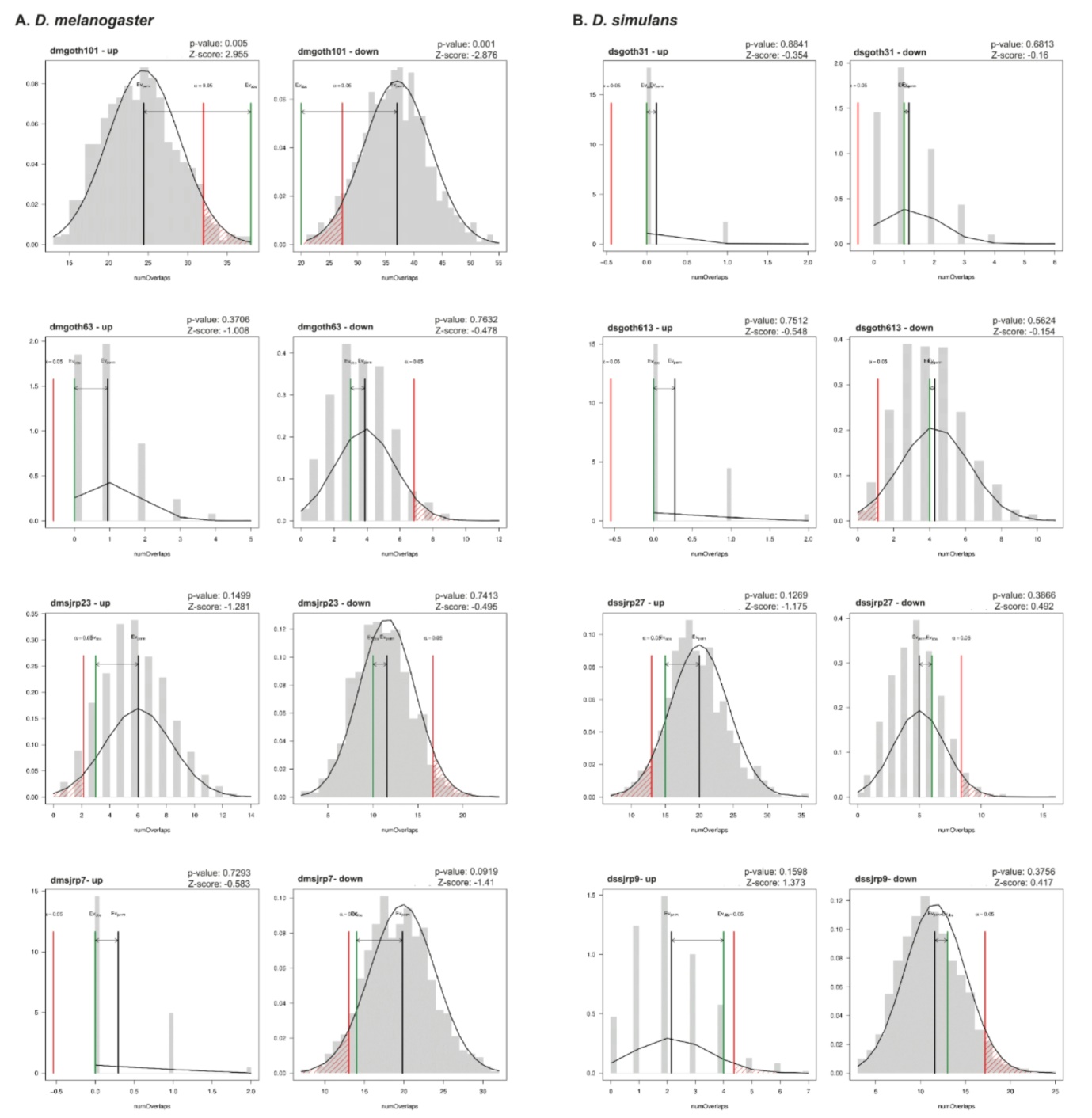



**Figure S10.** Results of the permutation test in all the strains tested after cold stress in **A)** *D. melanogaster* and **B)** *D. simulans*. Results of the permutation test in all the strains tested after starvation in **A)** *D. melanogaster* and **B)** *D. simulans*. To test whether TEs are enriched in the promoter regions of DEGs, we firstly created *bed* files with the 3 kb upstream region for these genes. Then, we performed permutation tests by intersecting TE positions with promoter regions using the R package *regioneR* (72), parameter *randomization = resampleRegions*, with 1000 random samples. Therefore, for each set of up and down-regulated genes with size *n*, the package identifies how many of them have TEs at the 3 kb upstream region. Finally, it performs 1000 samples of *n* genes randomly, to test whether the observed number of genes with TEs is greater or lower than expected in a genome-wide comparison. Histograms show the frequency of genes with TEs in the promoter region after 1000 random sampling. Black lines represent the expected values, red lines represent type I error ɑ level, and green lines represent the observed values. Significant results are found when the black and green lines are in opposite directions regarding the red line.

**
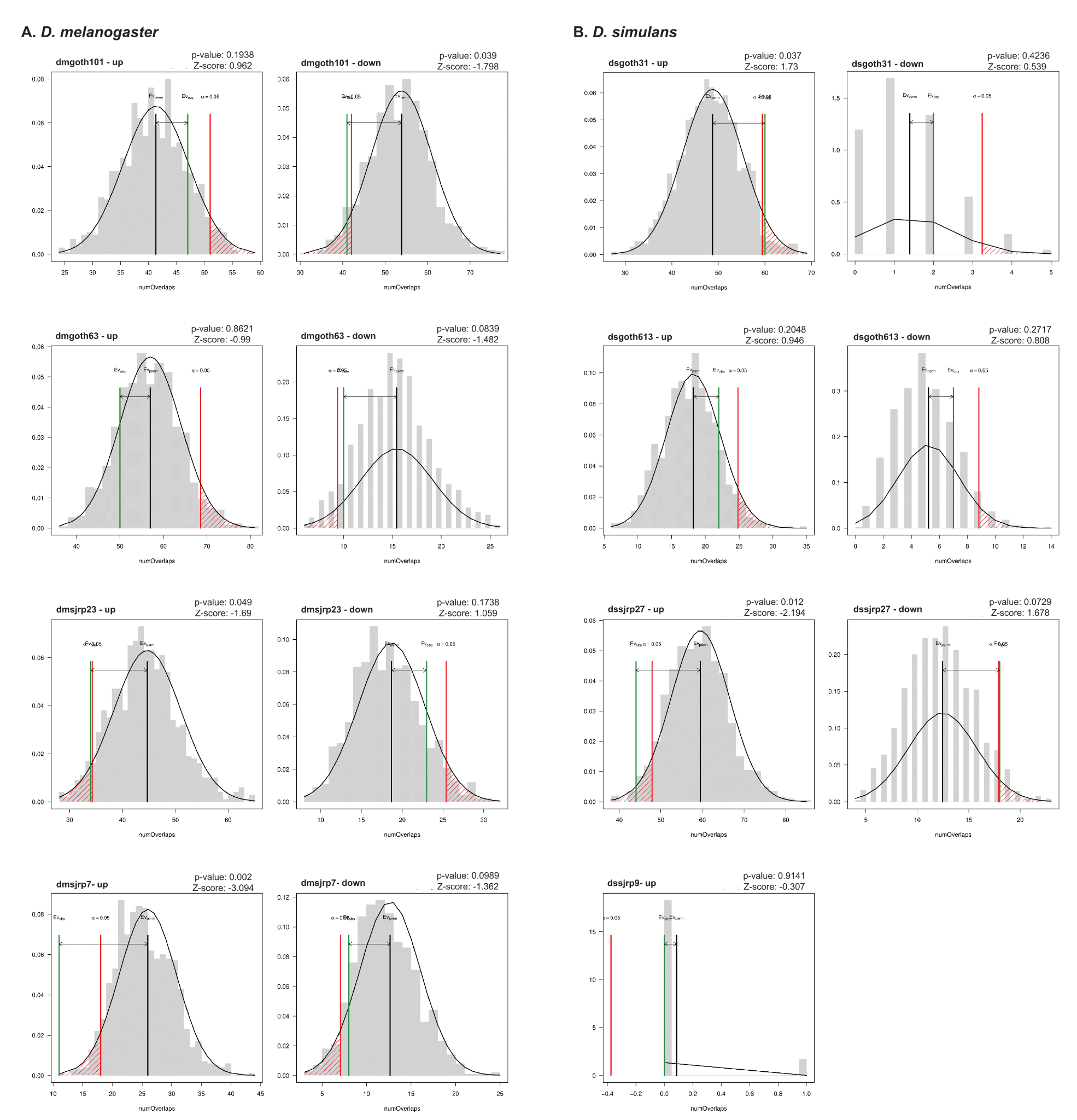
**

**Figure S11.** Scatter plot of the expression of TE families in both species and stress conditions. Scatter plots in starvation conditions in both **A**) *D. melanogaster* and **B**) *D. simulans*. Scatter plots after cold stress in both **C**) *D. melanogaster* and **D**) *D. simulan*s. Each dot represents a TE family. Up-regulated TE families are shown in red while down-regulated TE families are shown in blue, under the cutoffs of FDR < 0.05 and log2 fold-change > |1|.

**
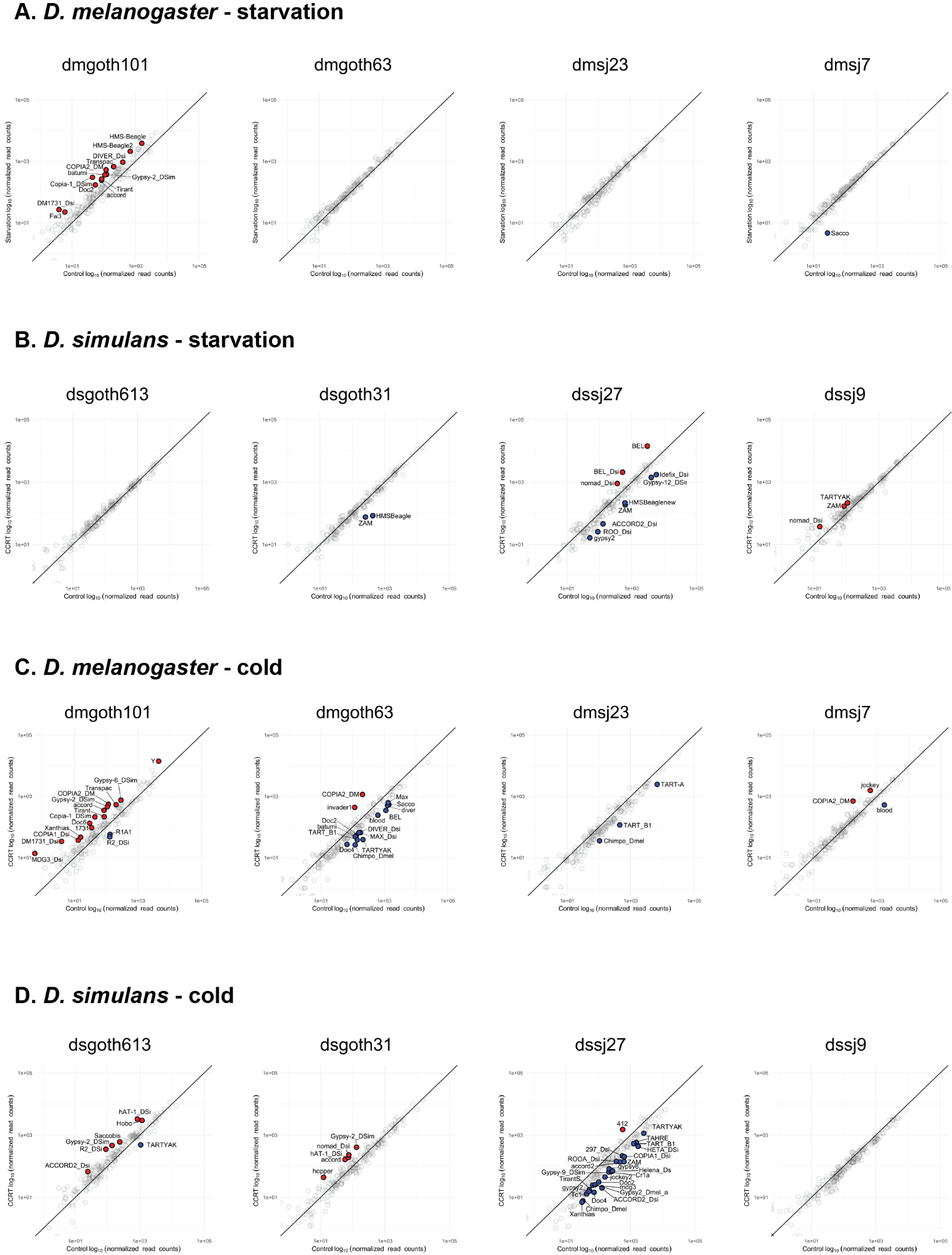
**

**Figure S12.** Variations in TE RNA counts and piRNA counts upon cold stress are independent in *D. melanogaster*. We tested whether there were statistical associations between the TE RNA variation and TE piRNA variation upon cold stress using Fisher exact tests at the TE family level. All four tests for *D. melanogaster* strains were not significant (p-values above each graph). Each dot represents a TE family.


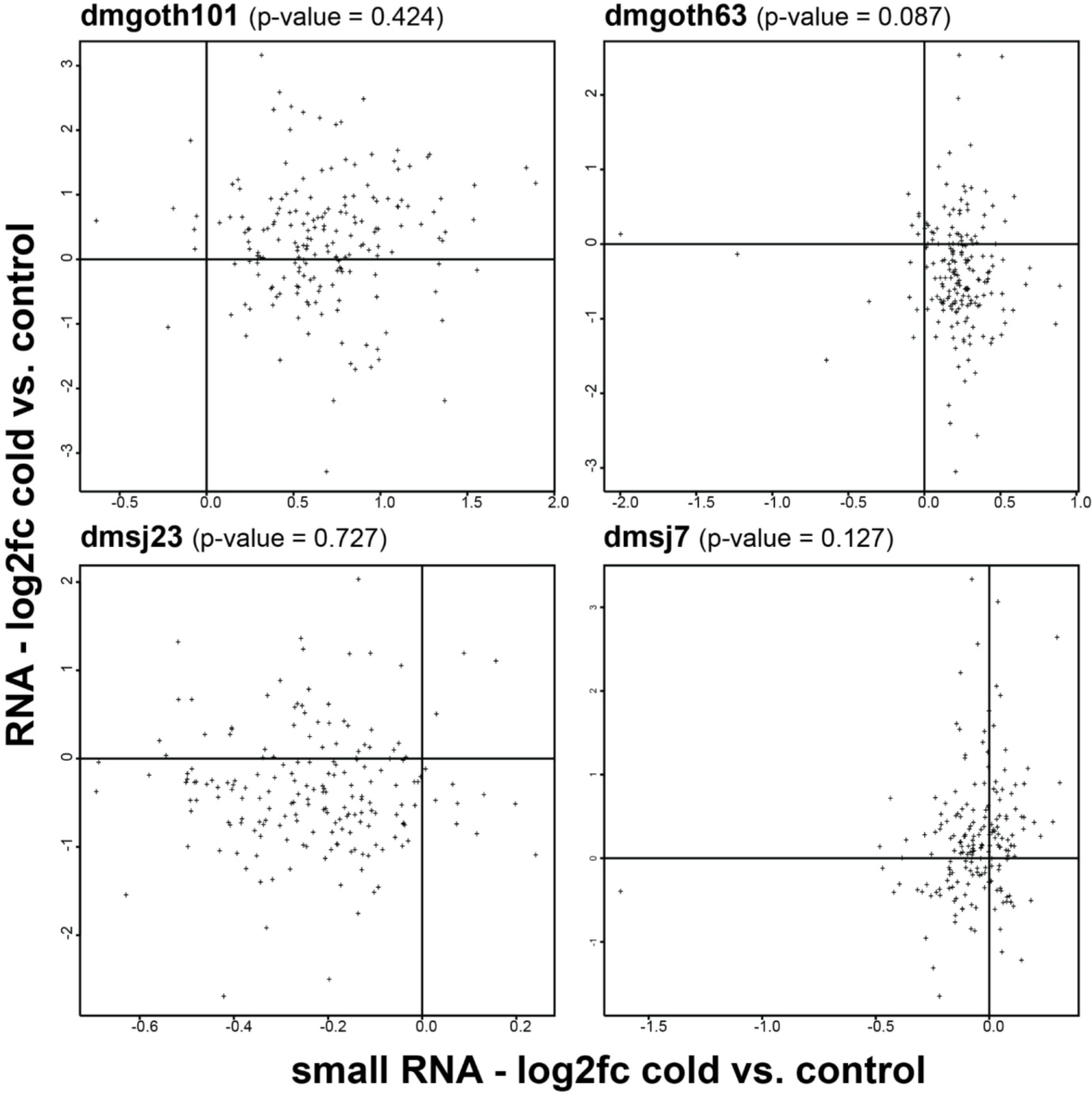



**Figure S13.** Distribution of ChIP-seq peaks near TSS regions in both **A**) *D. melanogaster* and **B**) *D. simulans*. For each condition and histone mark, two lines are depicted representing the two biological replicates used.

**Figure S14.** H3K4me3 and H3K9me3 profiling on 2 kb flanking regions of all TE insertions in **A)** *D. melanogaster* and **B)** *D. simulans* strains. For each condition and histone mark, two lines are depicted representing the two biological replicates used.

**Figure S15**. Histone mark profiles in the flanking regions of *D. melanogaster* TEs divided in DNA, LINE and LTR elements. Heatmap scale is based on normalization by RPGC (reads per genome content).




**Figure S16.** Histone mark profiles in the flanking regions of *D. simulans* TEs divided in DNA, LINE and LTR elements. Heatmap scale is based on normalization by RPGC (reads per genome content).




**Figure S17.** Upset graphs with shared chimeric transcripts between experimental conditions in **A)** *D. melanogaster* and **B**) *D. simulans*.


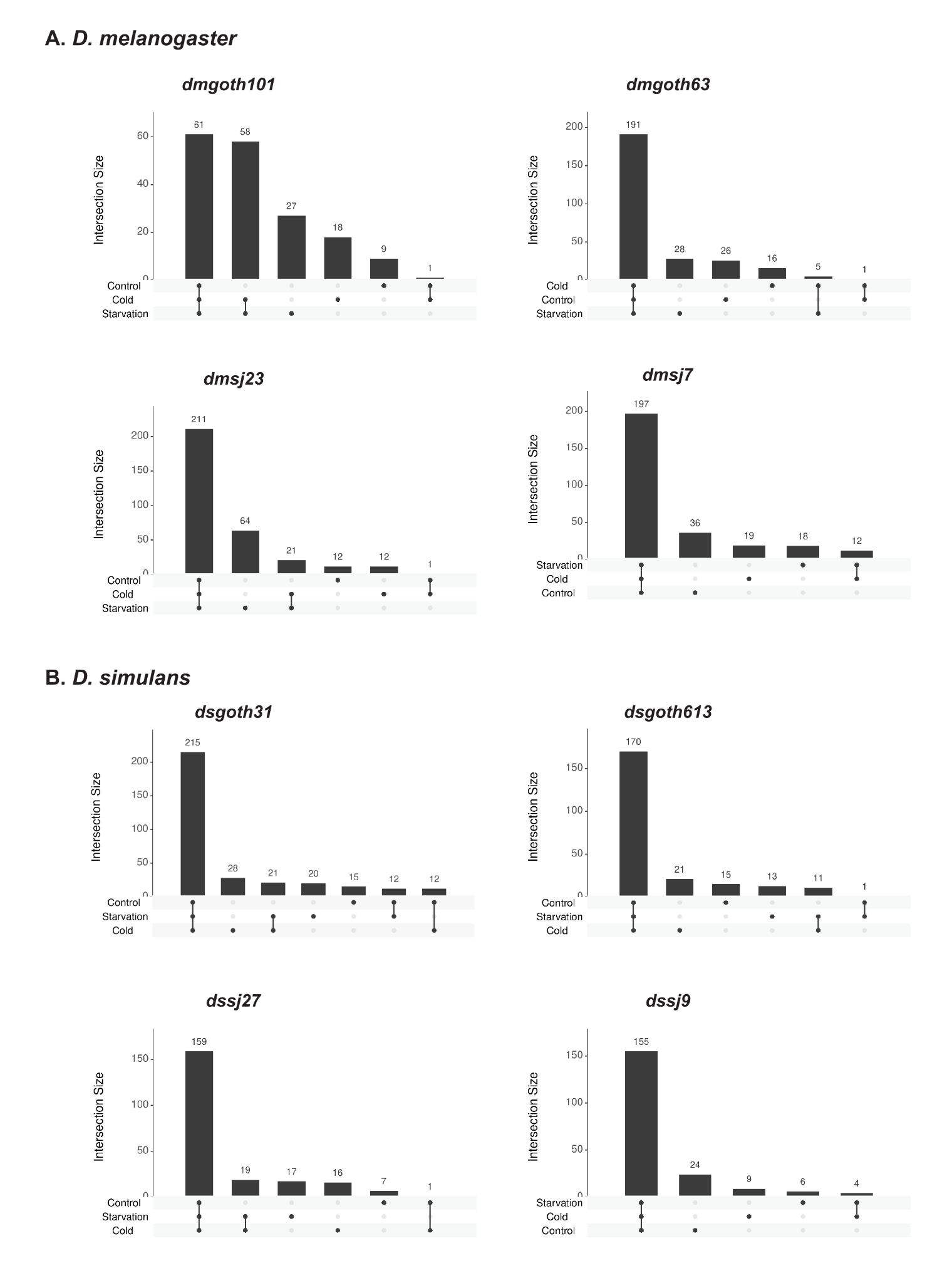


**Figure S18.** Pearson correlations between the TE insertion abundance in the genome for each family and the number of generated chimeric transcripts in **A**) *D. melanogaster* and **B**) *D. simulans* strains.


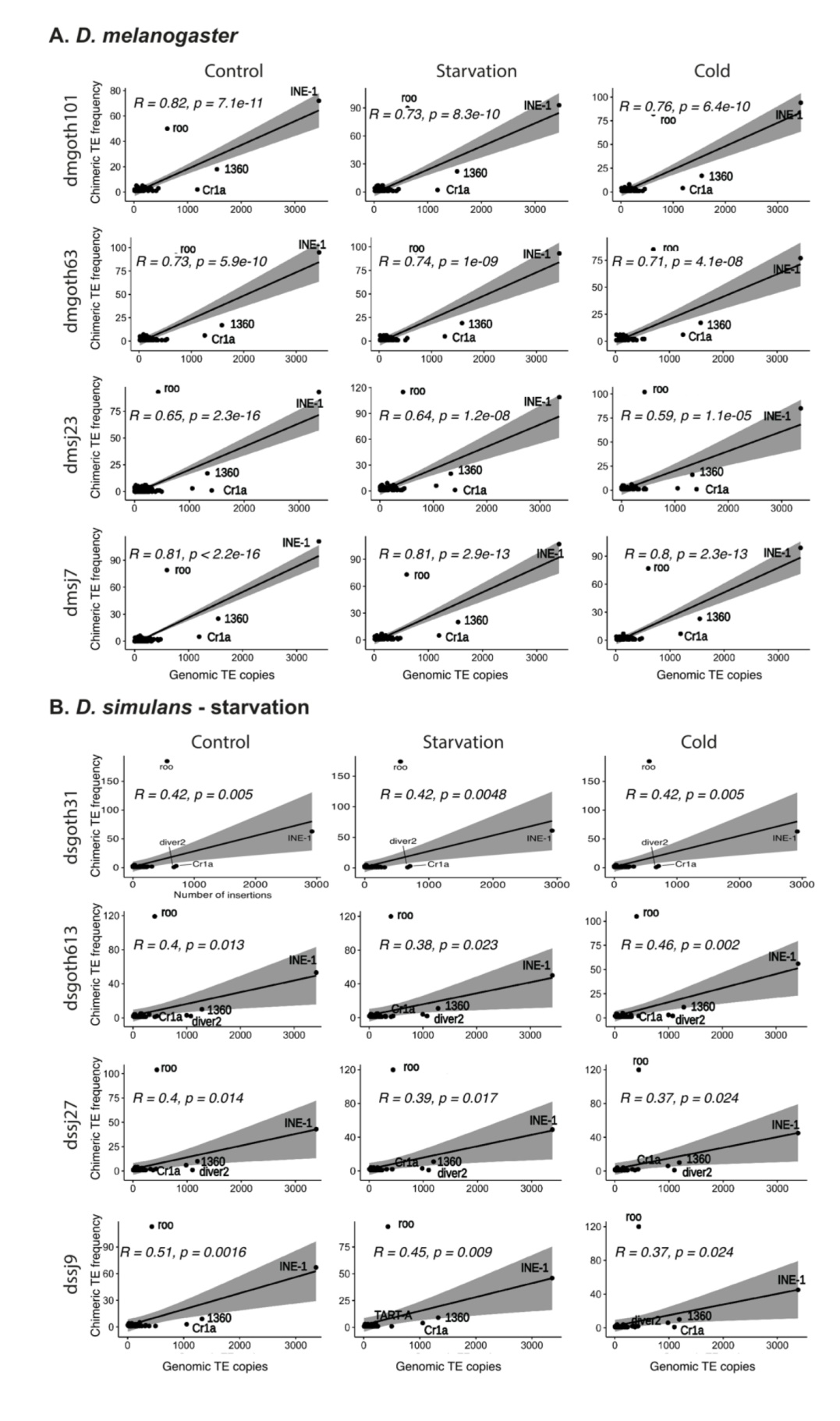

Supplement: Supplementary file 8 — Supplementary Material 8 [file 13100_2025_372_MOESM8_ESM.docx]
